# Determining Environmental Factors That Influence the Occurrence of Sarcoptic Mange in Bare-Nosed Wombats (*Vombatus ursinus*) Using Citizen Science Data

**DOI:** 10.1155/tbed/6264097

**Published:** 2025-12-03

**Authors:** Emily R. Fryett, Chandima N. Subasinghe, Julie M. Old, Hayley J. Stannard

**Affiliations:** ^1^School of Agricultural, Environmental and Veterinary Sciences, Charles Sturt University, Wagga Wagga, New South Wales, Australia; ^2^Spatial Data Analysis Network, Charles Sturt University, Albury, New South Wales, Australia; ^3^School of Science, Western Sydney University, Hawkesbury, New South Wales, Australia

**Keywords:** Australia, disease, marsupial, model, parasite, *Sarcoptes scabiei*

## Abstract

Sarcoptic mange is a significant welfare and conservation issue in a diverse range of mammalian species, wombats being some of the most impacted. Cases have been observed in bare-nosed (*Vombatus ursinus*) and southern hairy-nosed (*Lasiorhinus latifrons*) wombats but not the northern hairy-nosed wombat (*Lasiorhinus krefftii*). Here, we map the prevalence and model habitat suitability for sarcoptic mange that infests wombats across Australia. Presence-only data was isolated from wombat observation records entered into the citizen science project Wombat Survey and Analysis Tool (WomSAT). Environmental variables were collated from publicly available databases and prepared for analysis in ArcGIS Pro. Maxent modelling software was used to generate the habitat suitability model for wombat sarcoptic mange infestations. Sarcoptic mange contributed to 26.2% of all observations and were most frequent in Victoria (VIC) followed by New South Wales (NSW). The most important factors (percent contribution) in our model were Interim Biogeographic Regionalisation for Australia (IBRA; 25.7%), land use (24.5%), maximum summer temperature (15.1%), rain in summer season (10.7%), Collaborative Australian Protected Areas Database (CAPAD; 7.6%) and the hourly relative humidity at 3 pm (7.3%). All remaining environmental variables had very low to zero effect on the model. Our predictive presence model identified 73 local government areas (LGAs) across NSW (*n* = 20; 79.27 km^2^), VIC (*n* = 27; 265.46 km^2^) and Tasmania (TAS; *n* = 26; 21.52 km^2^) that had ‘highly suitable' conditions and 79 that had ‘suitable' conditions (3467.12 km^2^) for sarcoptic mange occurrence. In Queensland (QLD), only one LGA had ‘suitable' (0.61 km^2^) habitat for sarcoptic mange and no areas that were ‘highly suitable'. SA had four LGAs that were ‘moderately suitable' (74 km^2^) for sarcoptic mange, while the remaining LGAs were ‘not suitable'. The habitat in the ACT was predominantly ‘not suitable' for sarcoptic mange. Predictive models, such as this can be used to help plan management strategies and support treatment efforts by carers.

## 1. Introduction

Sarcoptic mange, caused by the astigmatid mite *Sarcoptes scabiei*, has an extremely broad host range, with infestations documented in more than 150 different mammalian species across 12 orders and 39 families [1]. The distribution of sarcoptic mange has likely been facilitated by humans, resulting in spillover events [1–3]. Wildlife species affected include black bears (*Ursus americanus*) in North America [4], ibex (*Capra pyrenaica*) in Spain [5, 6] and raccoon dogs (*Nyctereutes procyonoides*) in Asia [7, 8]. Cases of sarcoptic mange have been recorded in Australia since the early 1800 s, with the most severe clinical manifestations occurring in bare-nosed wombats (*Vombatus ursinus*) [9].

Bare-nosed wombats are large, fossorial, nocturnal and grazing marsupials native to Australia [10]. They are distributed in temperate regions of south-eastern Australia usually consisting of sclerophyll forest but can be found in varied habitats, including scrub, coastal heath and modified landscapes [11]. Currently, they are present along the east coast of New South Wales (NSW), south-eastern South Australia (SA), south-eastern corner of Queensland (QLD) and throughout Victoria (VIC) and Tasmania (TAS) and classified as ‘least concern' on the IUCN Red List [12].

Wombats infested by *S. scabiei* experience parakeratotic mange, where clinical signs include alopecia, erythema, pruritus, pyoderma, skin lesions and thickening of epidermis [13–16]. Excessive metabolic demands resulting from mange infestations result in emaciation and reduced reproductive success [14, 16]. A sarcoptic mange outbreak has been associated with a wombat population decline of 94% in Narawntapu National Park, TAS [3]. At various sites in NSW and VIC, the prevalence of sarcoptic mange was previously estimated to be 15%–41% in bare-nosed wombat populations [9, 13, 17–19]. More recently, the prevalence of sarcoptic mange in bare-nosed wombats across Australia was estimated to be 32% based on submissions by citizen scientists (2015–2019) [20].

Recent distribution modelling found sarcoptic mange was distributed throughout the entire range of bare-nosed wombats [20]; however, the western region of TAS was the least favourable for sarcoptic mange [21]. Ringwaldt et al. [21] demonstrated a positive relationship between mange presence and wombat habitat suitability, and a negative relationship with distance to water and topographic roughness with mange suitability in TAS. Landscapes that have been heavily modified were the most suitable land use types, and areas containing shrubland have the highest percentage of sarcoptic mange [21]. Similarly and also in TAS, Burgess et al. [22] found a negative relationship between the proportion of remnant vegetation in habitat and prevalence of sarcoptic mange. Also, Mayadunnage et al. [20] found as the distance to urban areas decreased, the suitability for sarcoptic mange increased. In addition, as the level of rainfall increased, the presence of sarcoptic mange increased, while elevation, isothermality and precipitation seasonality had a minimal effect on the suitability for sarcoptic mange [20].

Spatial epidemiology aims to describe and determine spatiotemporal variation in infectious disease occurrence in relation to the surrounding environment [23]. Many epidemiological processes influenced by both abiotic and biotic conditions drive the distribution of and dynamics between hosts, parasite populations and vectors [23]. Geographic information system (GIS) tools are utilised to construct frameworks, generate disease distribution maps and determine causative relationships [23]. Observations of *S. scabiei* infestations collected by citizen scientists can be used to improve our understanding of the spatial epidemiology of sarcoptic mange in wombat populations [20]. The citizen science project Wombat Survey and Analysis Tool (WomSAT) [24] was created to map the distribution of wombats with and without mange across Australia. Our study aimed to construct a predictive presence model to predict areas where sarcoptic mange is most likely to occur and factors that influence occurrence using citizen science data uploaded in WomSAT.

## 2. Methods

### 2.1. WomSAT Database

WomSAT (https://www.womsat.org.au) is a citizen science project documenting 23,360 sightings to date (March 22, 2024) [24]. These sightings include burrows, dead and live wombats, scats and tracks. Records date back to January 2010 and include date and time of observation, GPS location, whether the wombat was alive or dead and perceived mange score. The perceived mange score entered by citizen scientists into the database ranges from 1 to 3, where 1 indicates no signs of mange, 2 mild mange and 3 severe mange. Reference images are provided on the WomSAT webpage to assist users to determine a sarcoptic mange score. If the user is still not confident or cannot reliably determine a sarcoptic mange score, they can select unknown. The option to supply supporting images is also available to users which can provide a greater level of case verification.

### 2.2. Record Filtering

Data were downloaded from WomSAT including all records from January 2010 to March 2024 into Microsoft Excel (version 2404) and then filtered. A total of 15,430 live/dead wombat observations were downloaded, and from those sightings, records with a mange score of 1 (no mange) or unknown were removed. Prior to removing records, supporting images and/or comments were manually checked, and if a photograph showed the wombat missing hair or having crusted skin, the record was retained for further analysis. Sightings assigned score of 2 (mild) or 3 (severe) for mange with a photograph or comments were manually screened and removed from analysis if the photograph showed a healthy animal or commented the animal appeared to be in good condition. Further images that showed signs of wombat/dog attacks or cases where mange could not be determined due to the state of decay (in the case of records of dead animals), were removed. The mange presence scores were not differentiated in the analysis and were considered together. Records were carefully examined for duplicates which were removed, for example, records with the same comment, date, GPS location, time and user (Figure S1).

The data filtering process left 3241 (26.2%) viable records for mapping mange occurrence and training the predictive presence model. A species factor was added to the dataset for the SA records to distinguish between bare-nosed and southern hairy-nosed wombat observations. A presence-only model was used because a ‘disease-free' wombat does not equate to the disease being absent from that location and a sarcoptic mange infection can only be definitively confirmed using a skin scraping [25]. The location accuracy of some data points was low when overlaid with the Australian country administrative boundary using ArcGIS Pro 3.2.2 [26]; hence, they were eliminated from further processing.

### 2.3. Modelling Sarcoptic Mange Distribution

To reduce overfitting of the model, the study area was limited to the known range of bare-nosed wombats, thus excluding the Northern Territory (NT) and Western Australia (WA) as bare-nosed wombats do not occur in these two states [10]. Since the filtered data utilised are presence-only records, Maxent 3.4.4 software [27] was used to construct spatial models. The logistic output from Maxent was interpreted as the probability of occurrence (the raw output transformed to an estimated probability of species presence) of mange-infested bare-nosed wombats [28]. Explanatory variables (environmental factors) were collected from freely available databases and matched to the dataset using ArcGIS Pro to create the predictive presence model (Table S1).

To build and set the model, we followed the tutorial prepared by Young et al. [29]. We omitted distance to urban areas and roads in the final version of the model (however, the distance to urban areas was included in the bias file) due to a potential sampling bias present in the data collected by citizen scientists. It is more likely data is collected near where people are present and travel when recorded by citizen scientists [30]. Environmental variables that had no effect (0% contribution to the model) on the full model were removed from the final version of the model discussed.

### 2.4. Preparation of Environmental Layers

Our presence data file was shaped using ArcGIS Pro 3.2.2 [26] into a raster format. Environmental layers were all snapped to the same extent of 90 m and projected to the same coordinate system (GDA 94–Albers) and prepared as ASCII files [31]. A finer resolution is recommended for increased accuracy for the regionality and specificity of a model. A resolution of 90 m was selected to balance the effect of introducing standard error from downscaling the climate data while producing a fine resolution model. The Pearson correlation coefficients for the environmental variables utilised during the modelling process were calculated in MATLAB [32] and are available in Table S2.

### 2.5. Preparation of Bias Files

Sampling bias present in a dataset may lead to overfitting a model, making it difficult to determine whether species observations reflect true habitat preferences or simply increased survey effort in those habitats. To minimise bias, we incorporated a bias file into our model based on reported sarcoptic mange infestations across locations (local government areas; LGAs) [33].

### 2.6. Model Setting and Output

To effectively evaluate the performance of models, we used the Maxent setting of 25 random test percentages. The setting randomly withholds a specified percentage of the presence data from model creation (training data) for use to reliably evaluate the model's performance. For our model, 25% of all sarcoptic mange records were randomly withheld from model creation to assess the performance [29]. By randomly withholding a portion of our data, we eliminate bias without requiring an additional data source for model assessment.

The sampling technique used for our occurrence points was conducted by using the subsampling replicate run type in Maxent. Subsampling creates a smaller representative dataset from the original that can be used to assess a model's performance created from the training set. Averaging the accuracy of the model in predicting the subsample across multiple runs provides insight into the model's reliability. The model was run 15 times, and the results were averaged to produce a single suitability model. We utilised 5000 iterations to adequately sample for convergence with a default setting of one for the regularisation multiplier [29]. Several iterations of the model incorporating various combinations of environmental variables were performed to generate the most robust model with the least amount of bias. A robust statistical model is one where the results are not highly sensitive to minor contamination in the dataset producing reliable and accurate predictions. The average omission and predicted area and area under receiver-operating characteristic curve (AUC) obtained from the outputs of each iteration of the predictive presence model were used to assess performance. Only the results from the most robust model are reported here.

The Maxent logistic output was used to identify highly suitable habitats for sarcoptic mange infestations and categorised locations in a predicted suitability condition. The model was created using subsampling techniques by assigning raster cells with habitat suitability values ranging from 0 to 1 based on the effect of the environmental variables by the averaging of runs. Locations with greater values increase predicted habitat suitability for sarcoptic mange occurrence. The thresholds for suitable habitat were determined using the 10-percentile training presence logistic threshold obtained from the model output and used to determine the discrete habitat suitability categories. This method of threshold determination was chosen as it accounts for possible errors present in large datasets which may be the case for the WomSAT records. The minimum threshold for our model was calculated to be 0.2568 which was used to create the three classes of suitability: ‘moderately suitable', ‘suitable' and ‘highly suitable'. Raster cells that fall within 0–0.2567 probability are not suitable, 0.2568–0.5135 probability are moderately suitable, 0.5136–0.7703 probability are suitable and > 0.7704 probability are highly suitable. The suitability of LGAs was determined as the highest predicted suitability level within that area using area suitability calculations conducted in ArcGIS Pro.

## 3. Results

### 3.1. Distribution of Sarcoptic Mange in Wombat Populations

Sarcoptic mange occurred throughout the range of bare-nosed wombats in the ACT, NSW, TAS and VIC, but was undetected in QLD. Reported sarcoptic mange infestations were most prevalent in VIC (*n* = 1547) followed by NSW (*n* = 1257) with low numbers of observations in the ACT (*n* = 31), SA (*n* = 13) and TAS (*n* = 393) (Figure S2). In SA, sarcoptic mange in bare-nosed and southern hairy-nosed wombats was limited to the south-east corner of the state (Figure S2). The total number of southern hairy-nosed wombats reported showing signs of sarcoptic mange was 14 individuals in SA; hence, the data were not further modelled.

Wombats with observable signs of sarcoptic mange reported in VIC were distributed throughout the eastern half of the state across 33 LGAs (Figure S3). Within NSW, bare-nosed wombats infested with sarcoptic mange were distributed along the entirety of the east coast in 47 LGAs (Figure S4). Sarcoptic mange occurred throughout TAS except for the Derwent Valley, Huon Valley and West Coast (Figure S5). The entirety of the ACT was considered its own LGA with all sarcoptic mange observations in the northern half of the area (Figure S6). The number of sarcoptic mange infections was extremely low in SA and limited to the VIC border where bare-nosed wombats occur (Figure S7).

### 3.2. Temporal Distribution

A generalised linear model was fitted to count data using season, month and year as predictors. Initial modelling using a Poisson distribution revealed significant overdispersion, with the variance of the count variable (481.35) far exceeding its mean (19.88) and a dispersion statistic of 18.8, indicating the Poisson model was not appropriate. We refit the model using a negative binomial distribution to account for overdispersion. The final model showed that year was found to have a statistically significant negative association with count (*β* = −0.0444, SE = 0.020, *z* = −2.22, *p*=0.026), suggesting a decline in counts over time, with 2015 having the highest number of mange records (*n* = 807; Table 1). Among the categorical predictors, November was the only month with a statistically significant effect (*β* = −0.5195, *p*=0.037), indicating lower counts relative to April (Figure S8). Other seasonal and monthly effects were not statistically significant.

### 3.3. Factors Influencing Sarcoptic Mange in Wombats

**Figure 1:**
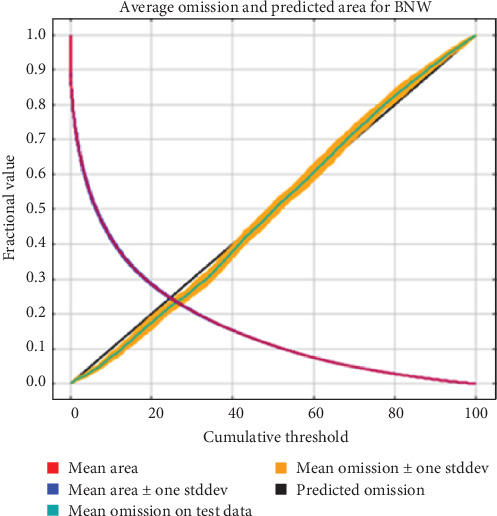
Average omission and predicted area for the bare-nosed wombat model to predict the suitability of *S. scabiei* generated in Maxent.

**Figure 2:**
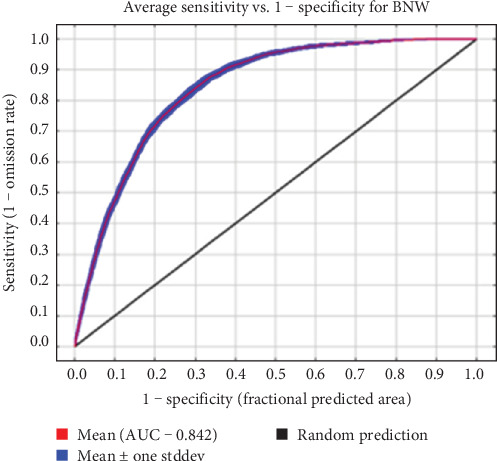
Area under receiver-operating characteristic curve for the bare-nosed wombat model to predict the suitability of *S. scabiei* generated in Maxent.

The average omission and predicted area, and AUC were used to assess model performance. The average omission measures whether a statistical model is missing important information. In a good model, the omission rate is close to the predicted omission rate with minimal variability [28, 29]. Since the model output in Figure 1 demonstrates a mean omission rate close to the predicted omission rate and displays minimal variability, it is deemed reliable. The AUC is the probability the model will correctly assess the presence of a site and rank them accordingly [28, 29]. The performance of a model with an AUC of 0.5 is no better than randomly fitting the data, while values closer to 1 indicate increased model performance reliability fitting the data better than random assignment [28, 29]. For our model, the average AUC for 15 replicate runs was 0.863 with a standard deviation of 0.006 (Figure 2) indicating a reliable model. The environmental variables present in the best-performing model are described in Table 2 as percent contribution and percent importance (proportion of unique information) [28, 29]. An environmental variable is considered important within the model if it contributes more than 5% of the predictive power of the model [29]. The most important environmental variables were Australia's bioregions (Interim Biogeographic Regionalisation for Australia; IBRA), land use, maximum summer temperature, rain in summer season, Collaborative Australian Protected Areas Database (CAPAD) and the hourly relative humidity at 3 pm (Table 2). All remaining environmental variables had very low (<1%) to zero effect on the model and were omitted from further analysis and discussion.

**Figure 3:**
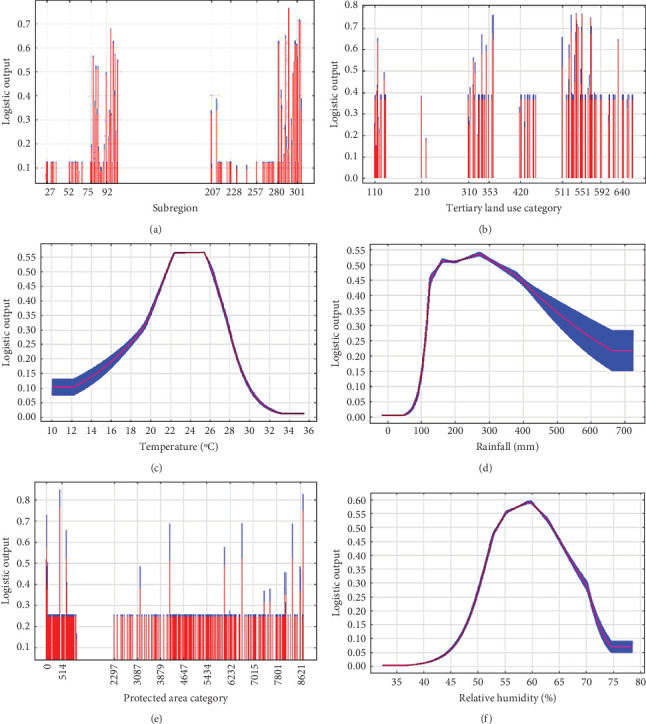
Response of sarcoptic mange suitability infesting bare-nosed wombats for (A) bioregion, (B) land use, (C) maximum summer temperature, (D) rain in summer, (E) protected areas and (F) relative humidity at 3 pm. Each graph shows the probability of mange presence across the range of each variable when reflecting the dependence of predicted suitability both on the selected variable and on dependencies induced by correlations between the selected variable and other variables. Red represents the mean of 15 model replicates, and blue is the standard deviation. Predicted probabilities between 0.51 and 0.76 on the environmental factor graphs are suitable, and probabilities above 0.77 are considered highly suitable for sarcoptic mange.

A total of 13 IBRA subregions from three bioregions in NSW, two bioregions in VIC (one of which occurs across NSW and VIC) and one bioregion in TAS were predicted to have a ‘suitable' probability of sarcoptic mange occurrence (Table 3, Figures 3A and S9). A total of 16 tertiary catchment-scale land use classifications had a ‘high probability' of being suitable for sarcoptic mange from four primary land use classifications (Figure 3B). Intensive land use had the greatest number of tertiary land use categories and the largest probability for sarcoptic mange, with the highest being urban residential (0.77) and the remaining described in Table 4. Production from dryland agriculture and plantations had the second highest number of tertiary land use classifications with the highest being degraded land (0.68) and the remaining described in Table 4. Conservation and natural environments was also a significant primary land use category (0.64) (Table 4). The final significant primary land use classification was water and plantations with the tertiary land use of river-intensive use (0.65) (Table 4).

The relationship between maximum summer temperature and habitat suitability demonstrated by the model starts low at 10°C (0.10) before steadily increasing to the suitable probability between 21.5 and 26°C, including the highest predicted probability of 0.57. There is a sharp decrease in prevalence after this range before stabilising at the lowest probability of 0.02 at 33°C (Figure 3C). The relationship between summer rainfall and habitat suitability demonstrated an initial low probability at 0 mm (0.01) before a sharp increase starting at 50 mm. The rainfall range predicted as suitable was between 155 and 320 mm with a maximum probability of 0.53 at 280 mm. Predicted habitat suitability slowly declines after this point until 650 mm (0.22) of rainfall before stabilising at higher levels of rainfall (Figure 3D).

The CAPAD regions that have the highest habitat suitability for sarcoptic mange, two nature reserves, one in NSW (0.78) and the other in the ACT (0.58); three natural feature reserves in VIC with predicted probabilities of 0.75 and two with 0.52 (Figure 3E).

The relationship between predicted habitat suitability and relative humidity started low at 32% (0.01), before exponentially increasing to the suitable probability between 54% and 64% humidity with a maximum probability at 59% (0.59). After this, steady decline in habitat suitability occurred until 74% humidity stabilising at 0.08 probability above this range (Figures 3F and S9).

### 3.4. Habitats Predicted to Have High Occurrence of Sarcoptic Mange

**Figure 4:**
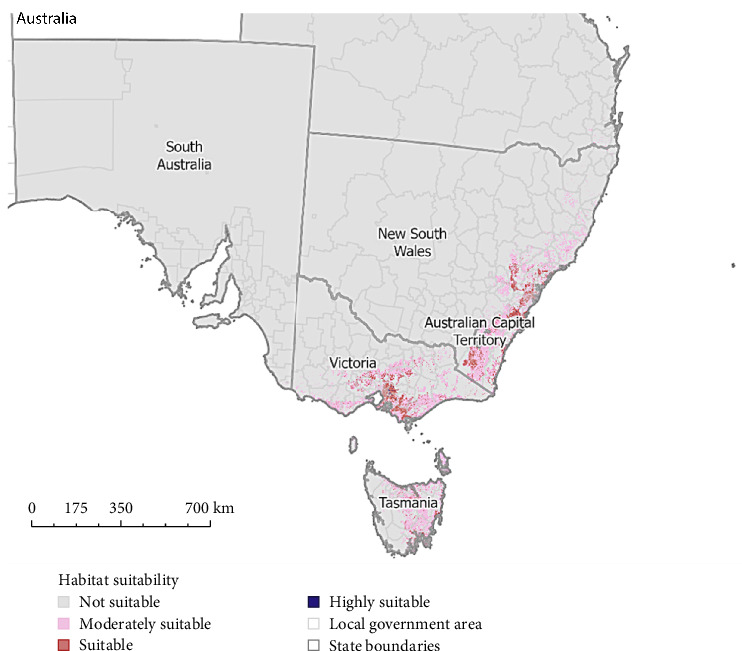
Continuous probability of the occurrence and suitability of sarcoptic mange in bare-nosed wombats. Raster cells with a predicted probability between 0 and 0.2567 were determined as not suitable for mange. Raster cells with a predicted probability between 0.2568 and 0.5135 were determined as moderately suitable for sarcoptic mange. Raster cells with a predicted probability between 0.5136 and 0.7703 were determined as suitable for sarcoptic mange. Raster cells with a predicted probability > 0.7704 were determined as highly suitable for sarcoptic mange.

**Figure 5:**
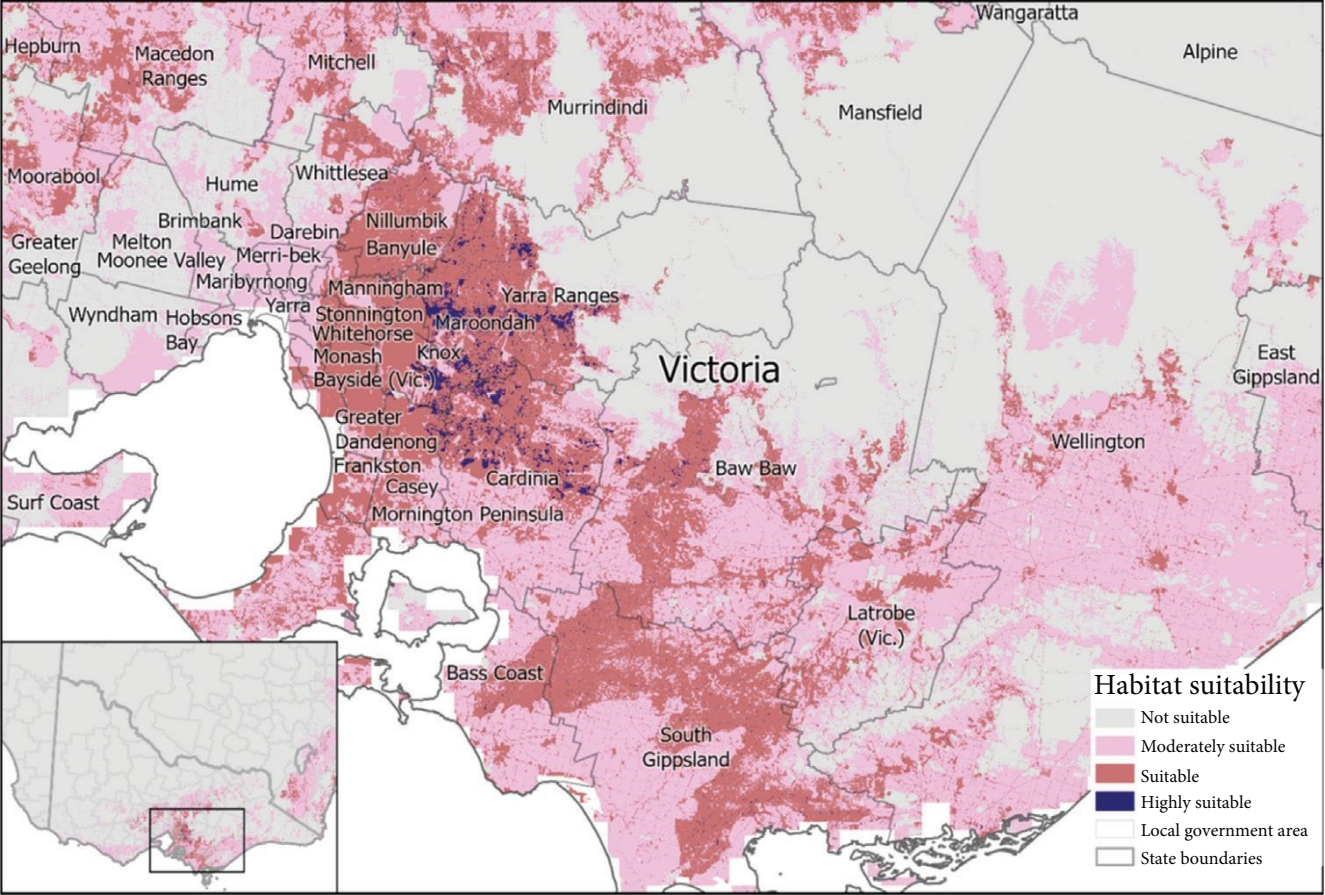
Continuous probability of the occurrence and suitability of sarcoptic mange in bare-nosed wombats located in Victoria.

**Figure 6:**
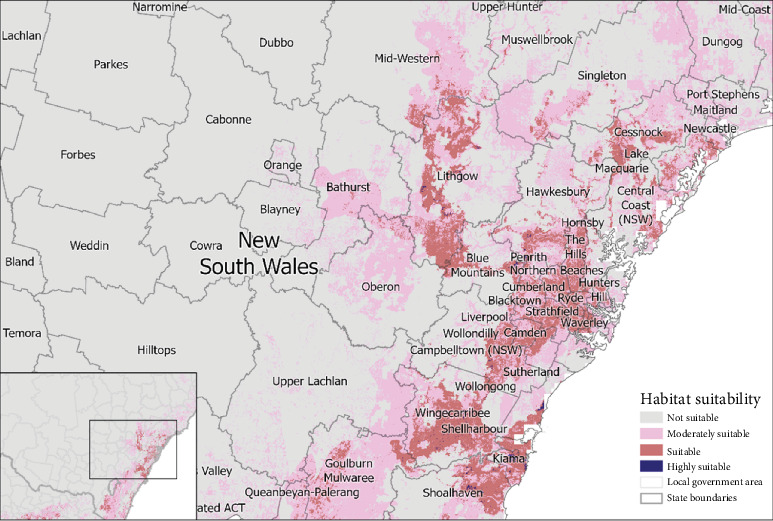
Continuous probability of the occurrence and suitability of sarcoptic mange in bare-nosed wombats located in New South Wales.

**Figure 7:**
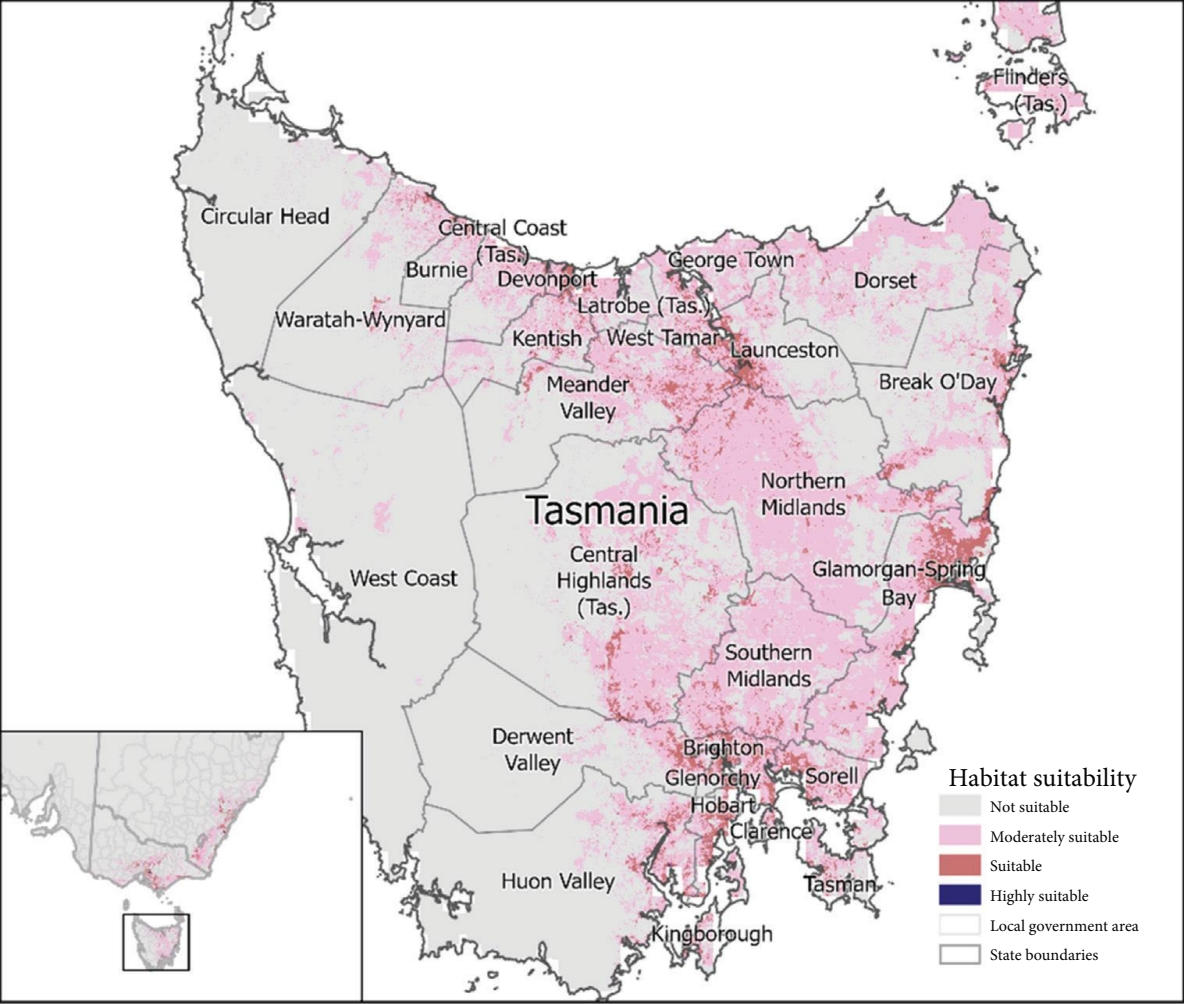
Continuous probability of the occurrence and suitability of sarcoptic mange in bare-nosed wombats located in Tasmania.

Our predictive presence model identified 73 LGAs that had ‘highly suitable' (>0.7704) areas and 79 ‘suitable' areas of habitat (0.5136–0.7703) for mange occurrence (Figure 4 and Table S3). The percentage of the total area of each LGA for each suitability category can be found in Table S4. In VIC, 27 LGAs had regions of ‘highly suitable' (265.46 km^2^), 29 had ‘suitable' (1240.78 km^2^) and 15 had ‘moderately suitable' (480.36 km^2^) and 8 were ‘not suitable' based on habitat for sarcoptic mange occurrence (Figure 5 and Table S3). In NSW, 20 LGAs had ‘highly suitable' (79.27 km^2^), 45 had ‘suitable' (2162.19 km^2^), 23 had ‘moderately suitable' (464.08 km^2^) and 40 were ‘not suitable' for sarcoptic mange occurrence (Figure 6 and Table S3). In TAS, 26 LGAs had ‘highly suitable' (21.52 km^2^) and three had ‘suitable' (59.72 km^2^) with no areas ‘moderately suitable' or ‘not suitable' (Figure 7 and Table S3).

In SA, four LGAs had ‘moderately suitable' (74.00 km^2^) habitat for sarcoptic mange (Table S3). All remaining areas were predicted to be ‘not suitable' for sarcoptic mange. Most of the ACT was predicted as ‘not suitable' for sarcoptic mange (Table S3). In QLD, only Scenic Rim was ‘suitable' (0.0001; 0.61 km^2^) for sarcoptic mange, while 24 LGAs had ‘moderately suitable' (1055.90 km^2^) habitat for sarcoptic mange occurrence. The remaining LGAs were ‘not suitable' (Table S3).

## 4. Discussion

Our study utilised the largest number of sarcoptic mange records in wombats across Australia to date and incorporated the largest collection of environmental variables into the modelling process. Sarcoptic mange infestations spanned almost the entire known range of bare-nosed wombats, only being absent in QLD and south-west TAS, but this may be due to low numbers of wombats reported to WomSAT. Sarcoptic mange was most prevalent in VIC and NSW along the eastern coastline while the ACT and SA had the lowest prevalence. There were 77 LGAs predicted to have a ‘high suitability' for sarcoptic mange infestations. The environmental variables that contributed the most to the model were bioregions, land use, maximum summer temperature, rain in summer season, protected areas and the hourly relative humidity at 3 pm.

### 4.1. Overall Sarcoptic Mange Prevalence

Of the total number of bare-nosed wombat observations recorded, 26.2% were infested with sarcoptic mange, which is lower than previously reported, 31.2% [20]. In our study, the highest number of sarcoptic mange records came from VIC (*n* = 1,547) compared to Mayadunnage et al. [20] who identified NSW as having the highest number of records. Our study included records from QLD and SA, spanned a longer time period and more records uploaded retrospectively for 2015 in VIC compared with Mayadunnage et al. [20] which may account for differences between prevalence reported in both studies.

### 4.2. Temporal Distribution

Our study found the reported occurrence of sarcoptic mange in wombats was statistically significant between seasons. There were significantly more cases of sarcoptic mange in winter compared to summer. Similarly, Hartley and English [17] reported that sarcoptic mange was more prevalent in wombats in winter (50%) compared to summer (22%). It has been speculated that low temperatures and high humidity support mite survival, or poor body condition of wombats contributes to the increase in sarcoptic mange observed in winter [13, 34]. Contrastingly, previous modelling using citizen scientist records failed to identify a significant effect of season on the occurrence of mange in bare-nosed wombats [20]. Previous studies in NSW and TAS using spotlighting or camera traps found that there was no significant difference in the occurrence of sarcoptic mange between seasons [19, 21, 22]. Stannard et al. [19] had smaller sample sizes and used spotlighting to determine mange occurrence, thus possibly lacked enough data to determine a seasonal influence.

There was a significantly higher number of sarcoptic mange records from 2015 compared to other years. WomSAT was established in 2015 [24] and likely attracted a higher number of users at that time compared to more recently. Data from 2010 to 2014 are retrospective records that users have entered; hence, they are reliant on people uploading stored data.

### 4.3. Factors Influencing Sarcoptic Mange

The IBRA environmental variable was the most important factor in predicting the habitat suitability for sarcoptic mange, which represents the general climate, geology, landform, native vegetation and species present in a given area [35]. For example, the South Eastern Highland bioregion spans VIC, NSW and the ACT and is categorised as having a temperate climate with areas of higher elevation experiencing a montane climate with milder summers [36]. The centring of high probability of sarcoptic mange suitability around areas of moderate summer temperatures with higher rainfall is in line with previous modelling of sarcoptic mange occurrence and mite survivability [20, 34, 37]. Other areas, such as the Sydney Basin bioregion, are located along the eastern coastline of NSW spanning from Nelson Bay in the north to Batemans Bay in the south, and as far west as Mudgee. The region is categorised as having a temperate climate with warm summers and lacking a dry season [36]. A high summer rainfall has been shown previously to support mange occurrence [20]. Based on the results of our model, the most suitable areas for sarcoptic mange to occur are areas that experience moderate temperatures, with mild summers, that are humid, and predominantly located along the eastern coastline of mainland Australia.

Land use was an important variable which may indicate the quality of habitat. Intensive land use may result in the alteration of diversity and number of vegetation communities leading to the degradation of surrounding habitat [38]. In particular, favouring communities with a higher abundance of introduced flora, due to the greater number of disturbance specialists when compared to native vegetation [38]. While wombats have been found in degraded habitats [39], they do not always have sufficient resources, for example, food or soil for burrows [10, 40]. Increased environmental pressures resulting from degraded habitats may increase stress to animals making them more susceptible to disease. Thus, sarcoptic mange is more likely to be prevalent in areas of degraded habitat which often lie in intensively used areas. Previous modelling by Burgess et al. [22] in TAS found as the proportion of exotic vegetation increased in surrounding habitat, the probability of mange also increased. They also found that as the amount of vegetation within the home range of a wombat decreased, the probability of mange increased [22]. Other modelling studies in TAS found a correlation between agricultural, exotic and urban areas and heavily modified for land use with sarcoptic mange [21, 41]. Our model produced similar results for the entire range of bare-nosed wombats with intensively used land and agricultural areas having a high probability to support sarcoptic mange. In addition, the proximity to domestic species within agricultural areas may also represent potential sources for novel infestation in wombats [42].

The maximum summer temperature was another significant variable in predicting sarcoptic mange habitat suitability with the highest probability between 21.5 and 26°C; however, wombats live in burrows that would have a different microclimate, for example, are able to maintain a stable temperature [34, 37, 43]. Previously, Ringwaldt et al. [21] found a moderate to high probability of sarcoptic mange occurrence associated with mean annual temperatures above 13°C.

Summer rainfall volume had a significant effect on the occurrence of sarcoptic mange, based on habitat suitability, with the highest probability between 155 and 320 mm. Likewise, previous studies found a positive correlation between the mange occurrence and rainfall [19, 20]. Ringwaldt et al. [21] found that annual rainfall > 800 mm and Mayadunnage et al. [20] that rainfall > 1400 mm reduced the probability of sarcoptic mange occurrence. It should be noted that previous studies used annual rainfall, whereas in our model, summer rainfall was significant.

Relative humidity at 3 pm was also a significant environmental variable in predicting the presence of sarcoptic mange. The highest probability of having suitable habitat for sarcoptic mange was between 54% and 64% humidity, suggesting it is an important factor in determining the habitat suitability for sarcoptic mange. Conditions of high humidity reduce the rate of desiccation of mites leading to increased survivability [34, 37]. Previous analysis by Stannard et al. [19] failed to identify a significant effect of humidity on mange prevalence, which may have been due to limited sample size.

### 4.4. Highly Suitable Habitats for Sarcoptic Mange in Bare-Nosed Wombats

Our study has reconfirmed the locations known to have sarcoptic mange present in the wombat population, identifying the same regions as Mayadunnage et al. [20]. LGAs with a high probability in our model that were not identified by Mayadunnage et al. [20] include Shellharbour and Snowy Monaro in NSW, and Casey, Knox, Macedon Ranges, Mitchell and Whittlesea in VIC. The difference between the areas predicted to have a high probability of suitable habitat in our model, and the previous model, could be the inclusion of the distance to roads and urban areas. We chose to omit these variables from our model and include them in the bias file.

The central north region of TAS was identified to have the highest prevalence of mange but is also known to occur in the central, east, northeast and northwest regions with no sarcoptic mange infestations reported in western TAS [41]. Our habitat suitability model identified similar trends with the Clarence, Devonport and Glenorchy regions having the highest predicted suitability when compared to Driessen et al. [41]. The lowest predicted habitat suitability was in the western region and the western half of the Derwent and Huon Valley regions. Similar results were obtained from a 5-year study conducted by Ringwaldt et al. [21] describing widespread but varied presence of mange across TAS, except the western region. The predictive model constructed by Mayadunnage et al. [20] also identified a high probability for suitable sarcoptic mange habitat in the central north and eastern regions of TAS, and low probability in the western region.

Our model predicted most of the ACT is ‘not suitable' (0.6886) for sarcoptic mange. However, a portion was predicted to be ‘moderately suitable' (0.3098) and ‘suitable' (0.0016), supported by previous studies (Table S3), only identifying one region that is likely to have sarcoptic mange present in the wombat population [13, 20]. Similarly, our model had low habitat suitability (0–0.2567) in SA and was supported by the survey by Martin et al. [13] that identified mange occurrence in five of eight localities.

## 5. Limitations

Citizen science has been used previously to monitor wildlife disease, determine impacts and determine environmental factors that influence disease presence [20, 44, 45]. However, a major limitation of using citizen science data is missing information within the records leading to over-/under-estimation of occurrence [46]. In the case of sarcoptic mange, this effect can be exaggerated by the difficulty of diagnosing early stages of infestation in wombats from observation alone [25]. Since the mean omission rate of our model was close to the predicted omission rate and displayed minimal variability, it indicates that our dataset is not missing major information required for a robust and reliable model despite these limitations.

The data used in our study are limited by accessibility, which potentially introduces bias into our model. People are likely to be reporting wombats near roads and urban regions. However, the inclusion of citizen scientist data has dramatically increased the overall sample size, study areas and duration of the records allowing for greater statistical power. We used a bias file for the distance between a mange observation and urban areas during the model creation process to address and counteract this effect while maintaining the statistical power granted by large-scale datasets collected by citizen scientists.

Our model is limited by the resolution of the climate variables being altered to match the rest of the environmental data (90 m). The spatial specificity of our model has been reduced; therefore, inferences made using this model should be made with caution. However the results are similar to previous modelling with the same but smaller dataset and likely representative of the trends of sarcoptic mange habitat suitability.

## 6. Conclusion

Our study demonstrates how long-term databases can be used to estimate the prevalence of sarcoptic mange in an animal population over large spans of space and time. Our study reinforces that sarcoptic mange in bare-nosed wombat populations is widespread throughout their known distribution. We described a seasonal effect of sarcoptic mange in wombat populations with winter having the greatest number of infestations reported. The IBRA, land use, maximum summer temperature, summer rainfall, CAPAD and relative humidity were all significant variables in the predictive model. Data should continue to be collected on unaffected and mange-affected wombats to capture changes in disease occurrence. Targeted monitoring of mange should be conducted in the field in areas predicted to have high habitat suitability to better understand trends in prevalence at the local scale. Our model can be used to support management plans and better support wildlife carers in treatment efforts.

## Figures and Tables

**Table 1:** Number of sarcoptic mange infested wombats reported to WomSAT in each year per state.

Year	ACT	NSW	SA	TAS	VIC	QLD	Total
2010	0	0	0	1	79	0	80
2011	0	22	0	0	95	0	117
2012	0	49	0	0	119	0	168
2013	0	48	0	0	115	0	163
2014	0	49	0	5	3	0	57
2015	12	320	5	51	419	0	807
2016	0	158	0	54	231	0	443
2017	4	84	1	186	221	0	496
2018	3	53	1	18	175	0	250
2019	3	119	1	38	34	0	195
2020	5	143	2	6	17	0	173
2021	2	88	2	20	20	0	131
2022	2	84	1	5	8	0	100
2023	0	38	2	8	10	0	57
2024	0	2	0	1	1	0	4
Total	31	1257	13	393	1547	0	3241

**Table 2:** Analysis of variable contributions for the best bare-nosed wombat model.

Variable	Description	Percent contribution	Percent importance
ibra	Interim biogeographic regionalisation for Australia	25.7	20.3
clum_50m1220m	Catchment scale land use of Australia (tertiary land use)	24.5	21.7
maxsum	1991–2020 Maximum summer temperature	15.1	3.6
rainsum	1991–2020 Average summer rainfall	10.7	13.6
capad_nodata_to0_mask1	Collaborative Australian protected areas database	7.6	2.6
rh15an1991_2020	1991–2020 Hourly relative humidity (3 pm)	7.3	6.9
clip_nvis06	Vegetation type	3.3	2
rainwet	1991–2020 Average wet season rainfall	2	1.5
distance_ahgf_minimum	Distance between water body and observation	1.2	0.8
90mready_clip_dem	Elevation (90 m)	0.9	15.3
rainwin	1991–2020 Average winter rainfall	0.8	0.6
rh9an1991_2020	1991–2020 Hourly relative humidity (9 am)	0.2	7
meanwin	1991–2020 Mean winter temperature	0.2	0.1
minwin	1991–2020 Minimum winter temperature	0.1	0.5
meanspr	1991–2020 Mean spring temperature	0.1	0.8
maxaut	1991–2020 Maximum autumn temperature	0.1	0.9
rainaut	1991–2020 Average autumn rainfall	0.1	0
maxoctapr	1991–2020 Maximum wet season temperature	0	1.6

**Table 3:** Interim Biogeographic Regionalisation for Australia (IBRA) and subregions predicted to have a 'suitable' (0.51–0.77) probability of sarcoptic mange occurrence.

IBRA bioregions	IBRA subregions	State	Predicted probability of suitable habitat for sarcoptic mange
South Eastern Highlands	Capertee uplands	NSW	0.76
	Bathurst	NSW	0.65
	Highlands-southern fall	VIC	0.67
	Strzelecki ranges	VIC	0.62
	Monaro	NSW/VIC	0.57

Sydney Basin	llawarra	NSW	0.71
	Moss vale	NSW	0.71
	Wyong	NSW	0.62
	Cumberland	NSW	0.61
	Yengo	NSW	0.54

Riverina	Lachlan	NSW	0.62

Victorian midlands	Central victorian uplands	VIC	0.55

Furneaux	Flinders	TAS	0.56

**Table 4:** Primary and associated tertiary land use categories determined to have the highest predicted probability of mange occurrence.

Primary land use	Tertiary land use	Probability of mange occurrence
Intensive land use	Urban residential	0.77
Intensive land use	Roads	0.75
Intensive land use	Rural areas with agriculture	0.72
Intensive land use	Rural areas without agriculture	0.71
Intensive land use	Farm buildings/infrastructure	0.69
Intensive land use	Major industrial complex	0.68
Intensive land use	Railways	0.68
Intensive land use	Water extraction and transmission	0.57
Intensive land use	Abandoned intensive animal production	0.56
Production from dryland agriculture and plantations	Degraded land	0.68
Production from dryland agriculture and plantations	Abandoned land	0.65
Production from dryland agriculture and plantations	Seasonal horticulture	0.59
Production from dryland agriculture and plantations	Tree nuts	0.58
Production from dryland agriculture and plantations	Native/exotic mosaic	0.55
Conservation and natural environments	Protected landscapes	0.64
Water and plantations	River-intensive use	0.65

## Data Availability

The data that support this study are available in the article and accompanying online supporting information section.

## Supporting Information

Supporting Information Figure S1. Data filtering process showing the total number of records and those removed from the WomSAT data to reach the final records that were included in the analysis. Figure S2A. National map of bare-nosed and southern hairy-nosed wombats showing observable signs of sarcoptic mange. B. National heatmap of bare-nosed and southern hairy-nosed wombats showing observable signs of sarcoptic mange. Figure S3A. Map of bare-nosed wombats within VIC showing observable signs of sarcoptic mange. B. Heat map of bare-nosed wombats within VIC showing observable signs of sarcoptic mange. Figure S4A. Map of bare-nosed wombats within NSW showing observable signs of sarcoptic mange. B. Heat map of bare-nosed wombats within NSW showing observable signs of sarcoptic mange. Figure S5A. Map of bare-nosed wombats within TAS showing observable signs of sarcoptic mange. B. Heat map of bare-nosed wombats within TAS showing observable signs of sarcoptic mange. Figure S6A. Map of bare-nosed wombats within the ACT showing observable signs of sarcoptic mange. B. Heat map of bare-nosed wombats within the ACT showing observable signs of sarcoptic mange. Figure S7A. Map of bare-nosed (BNW) and southern hairy-nosed (SHNW) wombats in South Australia showing observable signs of sarcoptic mange. B. Heat map of bare-nosed wombats within SA showing observable signs of sarcoptic mange. Figure S8. Seasonal distribution of sarcoptic mange in wombats reported to WomSAT between 2010 and 2024. Figure S9. Response of sarcoptic mange suitability infesting bare-nosed wombats for A, bioregion; B, land use; C, maximum summer temperature; D, rain in summer; E, protected areas; F, relative humidity at 3 pm. Each graph shows the probability of mange presence across the range of each variable when reflecting the dependence of predicted suitability both on the selected variable and on dependencies induced by correlations between the selected variable and other variables. Red represents the mean of 15 model replicates, and blue is the standard deviation. Predicted probabilities between 0.51 and 0.76 on the environmental factor graphs are suitable, and probabilities above 0.77 are considered highly suitable for sarcoptic mange. Green dots represent observed data as a percentage. Table S1. Environmental layer description and classification of data type used to create the model of habitat suitability for sarcoptic mange occurrence in bare-nosed wombats. Table S2. Pearson's coefficients for the environmental factors in the final bare-nosed wombat predictive presence model. Table S3. Predicted probability (% of total area) for sarcoptic mange habitat suitability for local government areas within New South Wales, South Australia, Tasmania, Queensland and Victoria.
