# Supplementary material for: Determining Environmental Factors That Influence the Occurrence of Sarcoptic Mange in Bare-Nosed Wombats (Vombatus ursinus) Using Citizen Science Data
Source: Transbound Emerg Dis. 2025 Dec 3;2025:6264097. doi: 10.1155/tbed/6264097 (PMC12695404; doi:10.1155/tbed/6264097)
Supplement: Supporting Information — Figure S1. Data filtering process showing the total number of records and those removed from the WomSAT data to reach the final records that were included in the analysis. Figure S2A. National map of bare-nosed and southern hairy-nosed wombats showing observable signs of sarcoptic mange. B. National heatmap of bare-nosed and southern hairy-nosed wombats showing observable signs of sarcoptic mange. Figure S3A. Map of bare-nosed wombats within VIC showing observable signs of sarcoptic mange. B. Heat map of bare-nosed wombats within VIC showing observable signs of sarcoptic mange. Figure S4A. Map of bare-nosed wombats within NSW showing observable signs of sarcoptic mange. B. Heat map of bare-nosed wombats within NSW showing observable signs of sarcoptic mange. Figure S5A. Map of bare-nosed wombats within TAS showing observable signs of sarcoptic mange. B. Heat map of bare-nosed wombats within TAS showing observable signs of sarcoptic mange. Figure S6A. Map of bare-nosed wombats within the ACT showing observable signs of sarcoptic mange. B. Heat map of bare-nosed wombats within the ACT showing observable signs of sarcoptic mange. Figure S7A. Map of bare-nosed (BNW) and southern hairy-nosed (SHNW) wombats in South Australia showing observable signs of sarcoptic mange. B. Heat map of bare-nosed wombats within SA showing observable signs of sarcoptic mange. Figure S8. Seasonal distribution of sarcoptic mange in wombats reported to WomSAT between 2010 and 2024. Figure S9. Response of sarcoptic mange suitability infesting bare-nosed wombats for A, bioregion; B, land use; C, maximum summer temperature; D, rain in summer; E, protected areas; F, relative humidity at 3 pm. Each graph shows the probability of mange presence across the range of each variable when reflecting the dependence of predicted suitability both on the selected variable and on dependencies induced by correlations between the selected variable and other variables. Red represents the mean of 15 model replicat [file 6264097.f1.docx]

**Supplementary Materials**

**
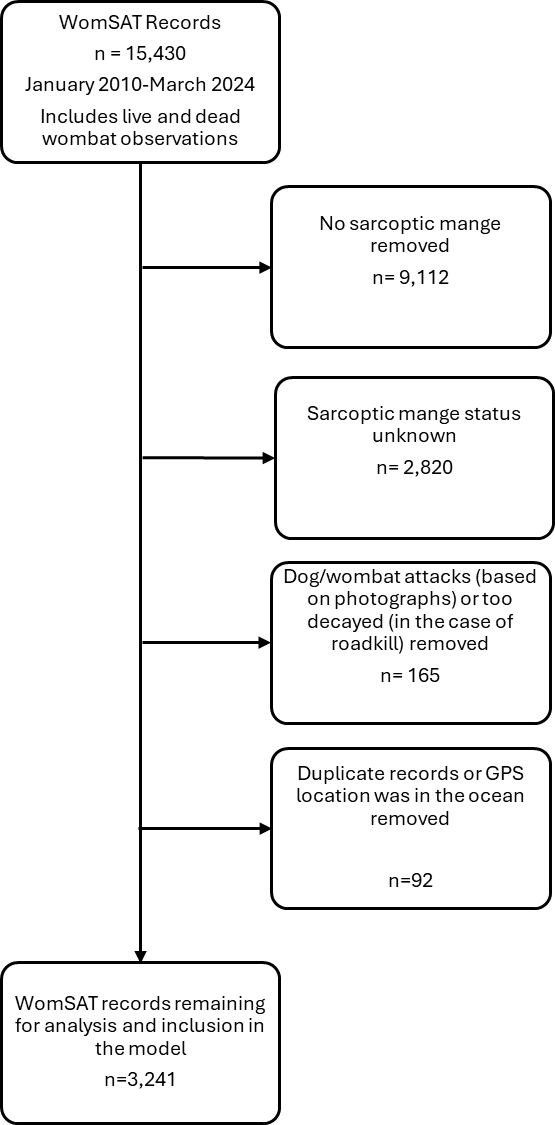
**

Supplementary Figure 1. Data filtering process showing the total number of records and those removed from the WomSAT data to reach the final records that were included in the analysis.


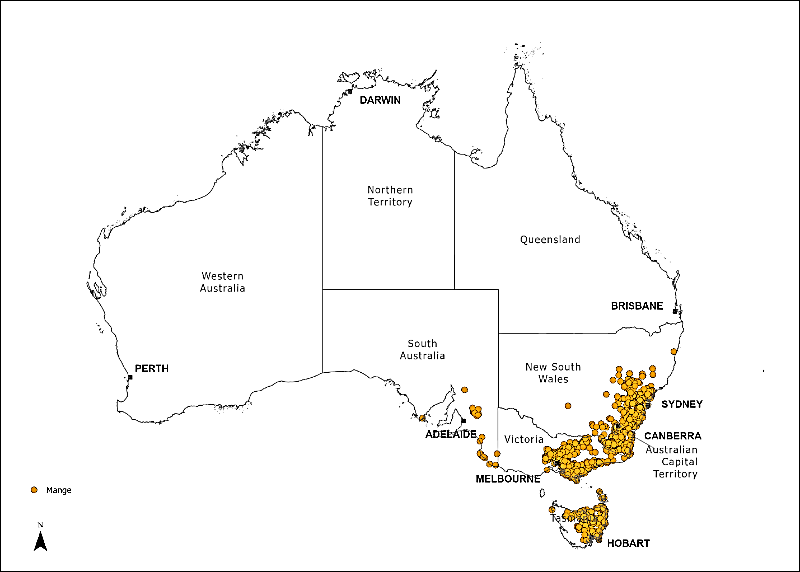

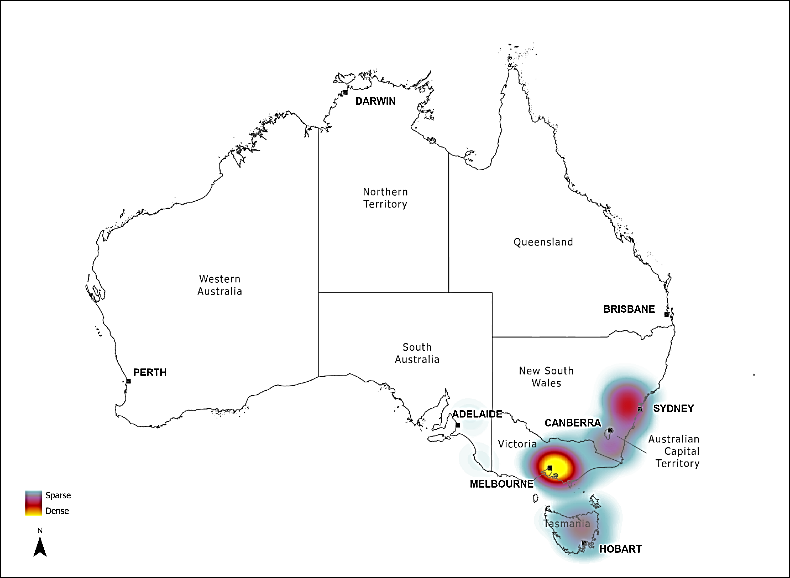

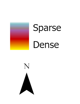

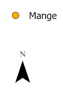


A

B

Supplementary Figure 2A. National map of bare-nosed and southern hairy-nosed wombats showing observable signs of sarcoptic mange. B. National heatmap of bare-nosed and southern hairy-nosed wombats showing observable signs of sarcoptic mange.


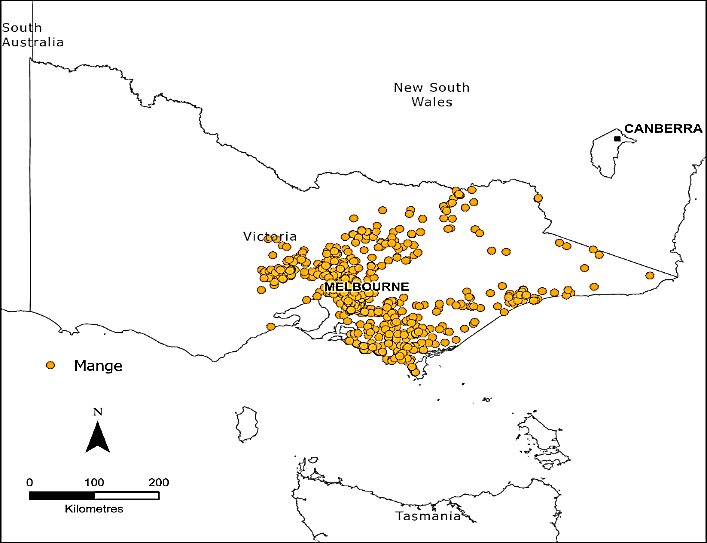

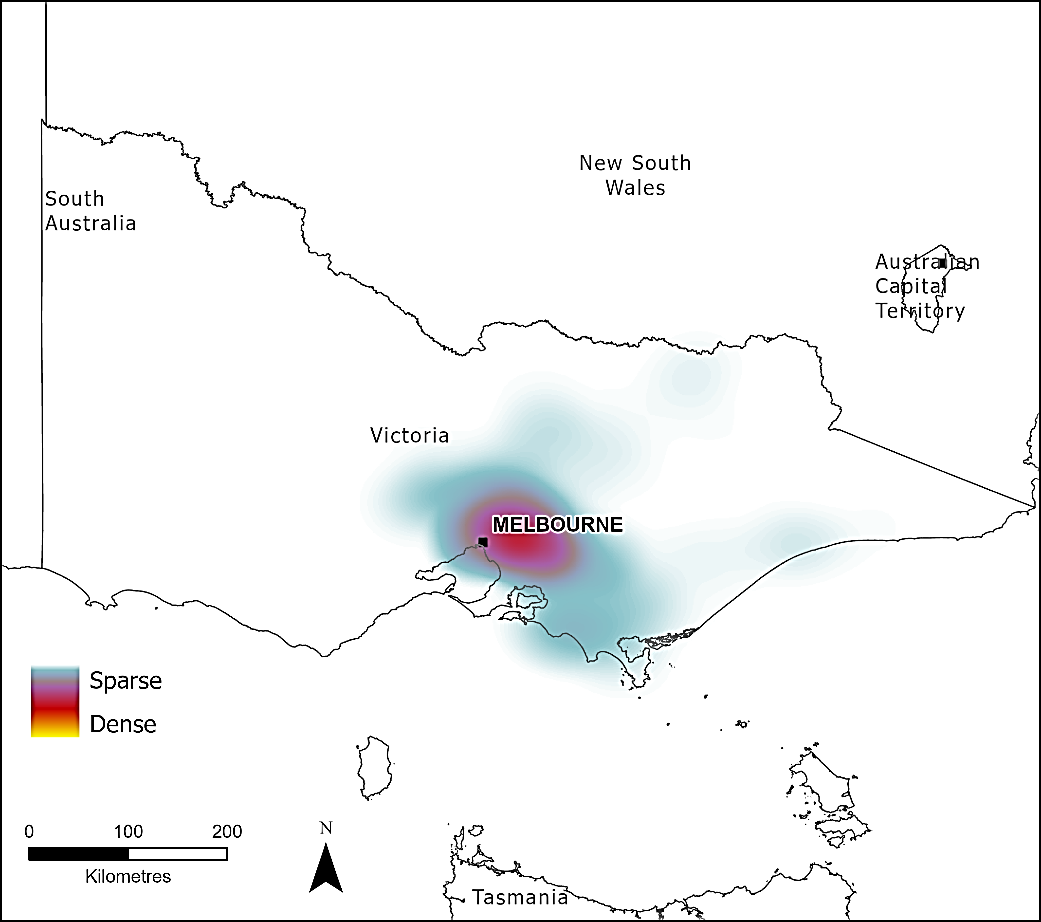


A

B

Supplementary Figure 3A. Map of bare-nosed wombats within VIC showing observable signs of sarcoptic mange. B. Heat map of bare-nosed wombats within VIC showing observable signs of sarcoptic mange.


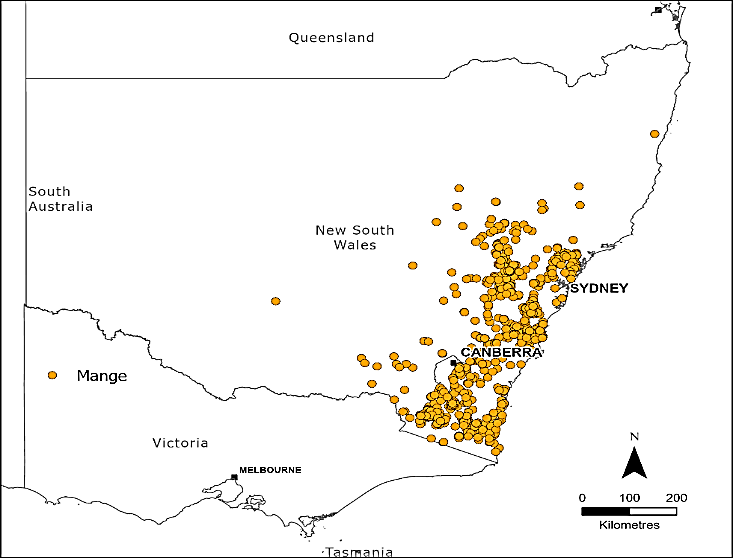

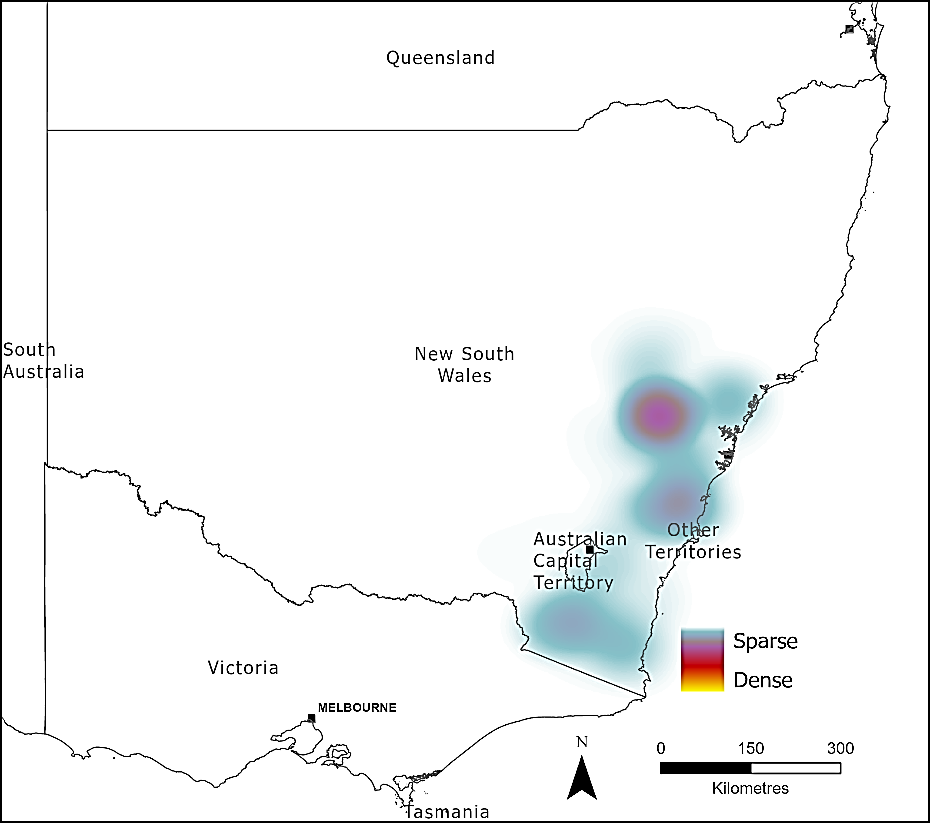


A

B

Supplementary Figure 4A. Map of bare-nosed wombats within NSW showing observable signs of sarcoptic mange. B. Heat map of bare-nosed wombats within NSW showing observable signs of sarcoptic mange.


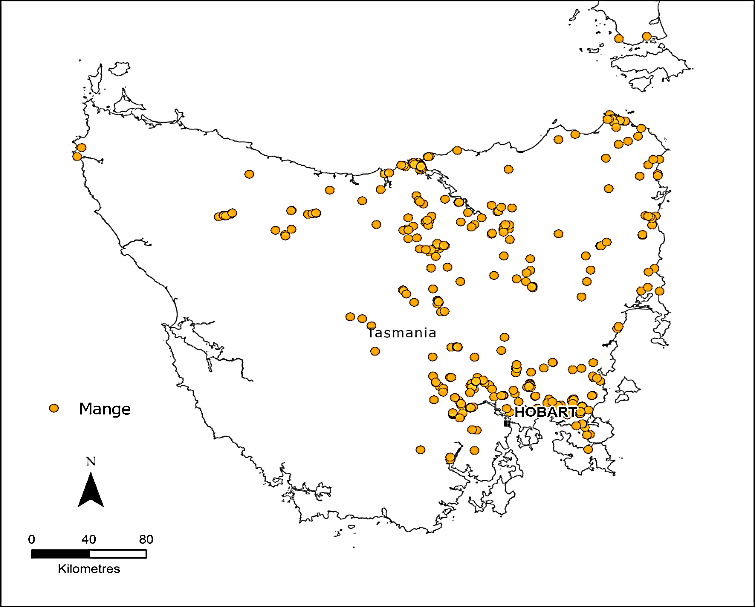

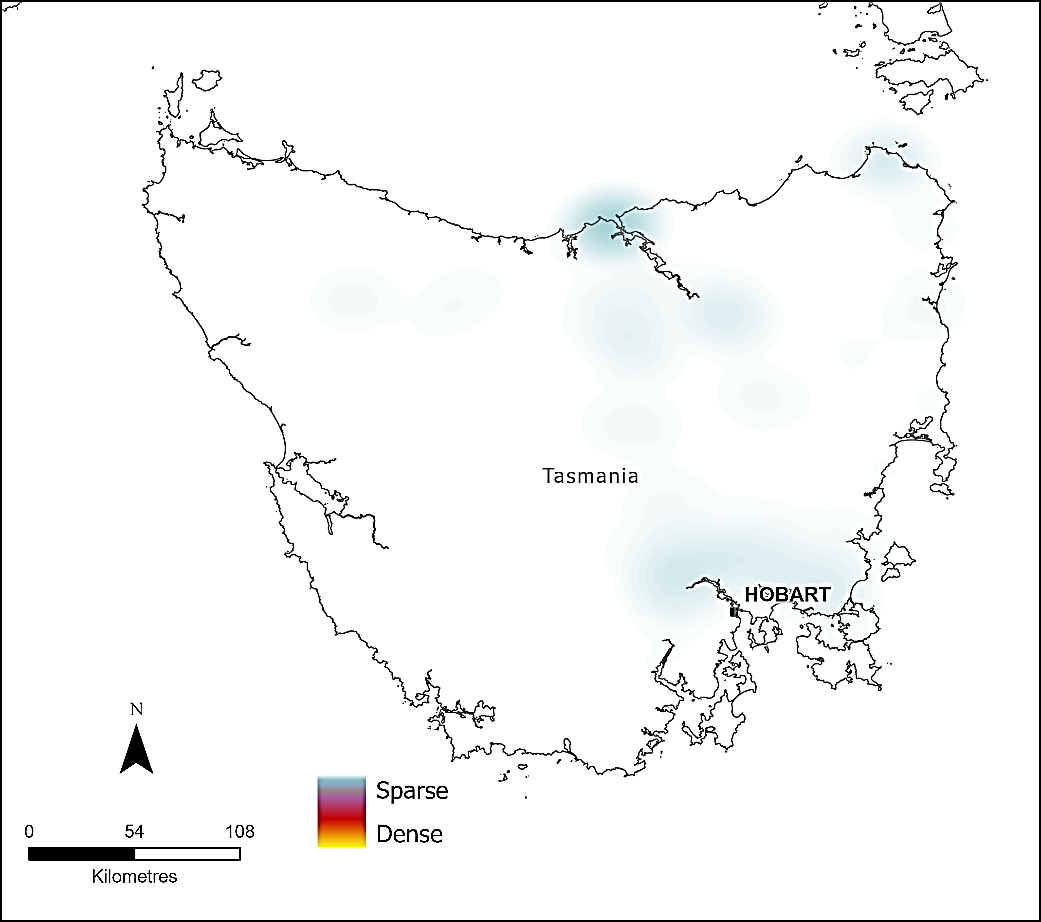


A

B

Supplementary Figure 5A. Map of bare-nosed wombats within TAS showing observable signs of sarcoptic mange. B. Heat map of bare-nosed wombats within TAS showing observable signs of sarcoptic mange.


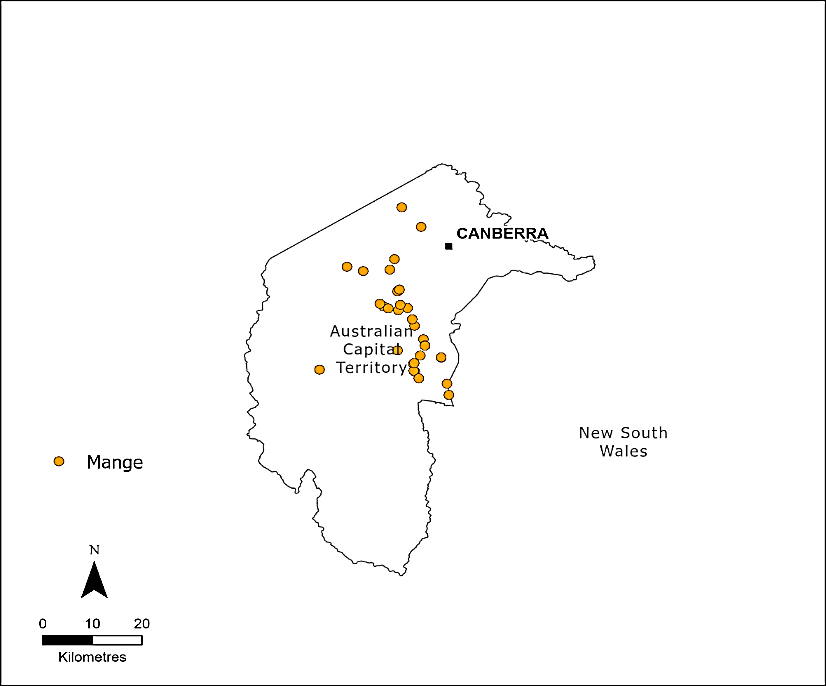

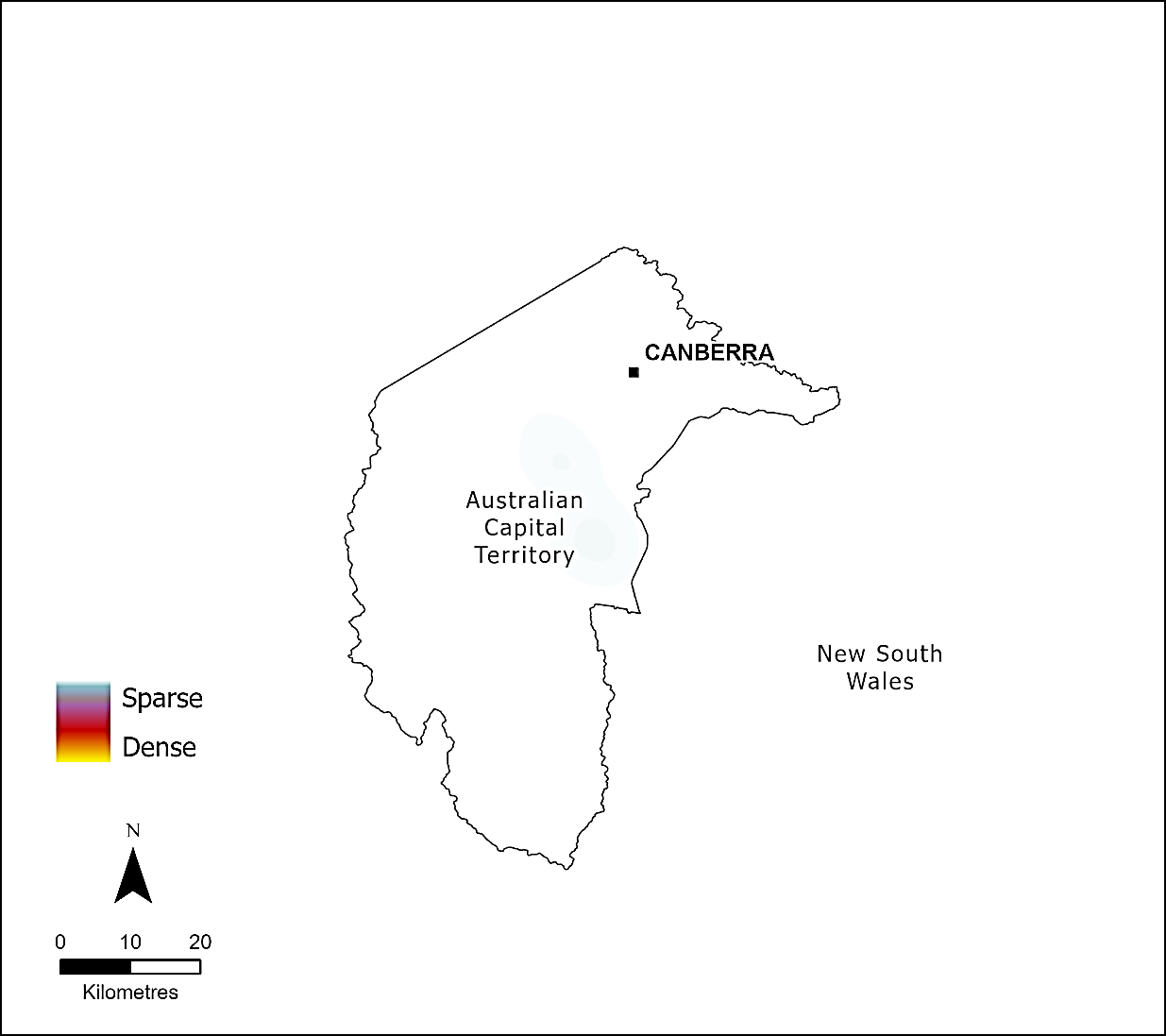


A

B

Supplementary Figure 6A. Map of bare-nosed wombats within the ACT showing observable signs of sarcoptic mange. B. Heat map of bare-nosed wombats within the ACT showing observable signs of sarcoptic mange.


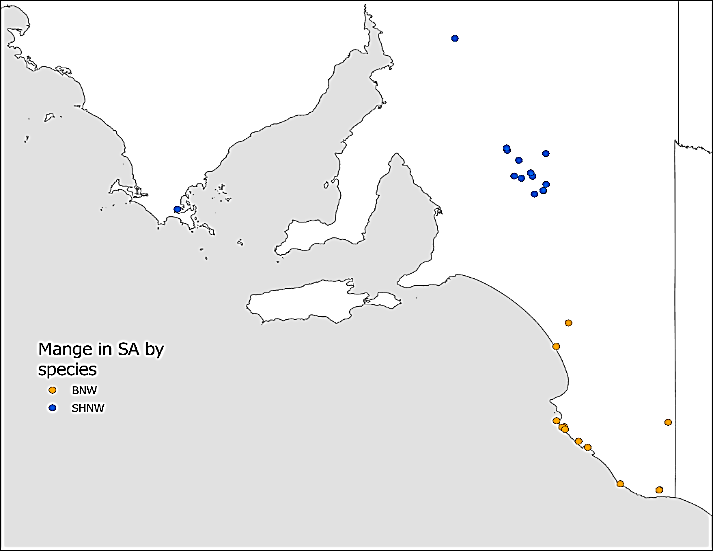

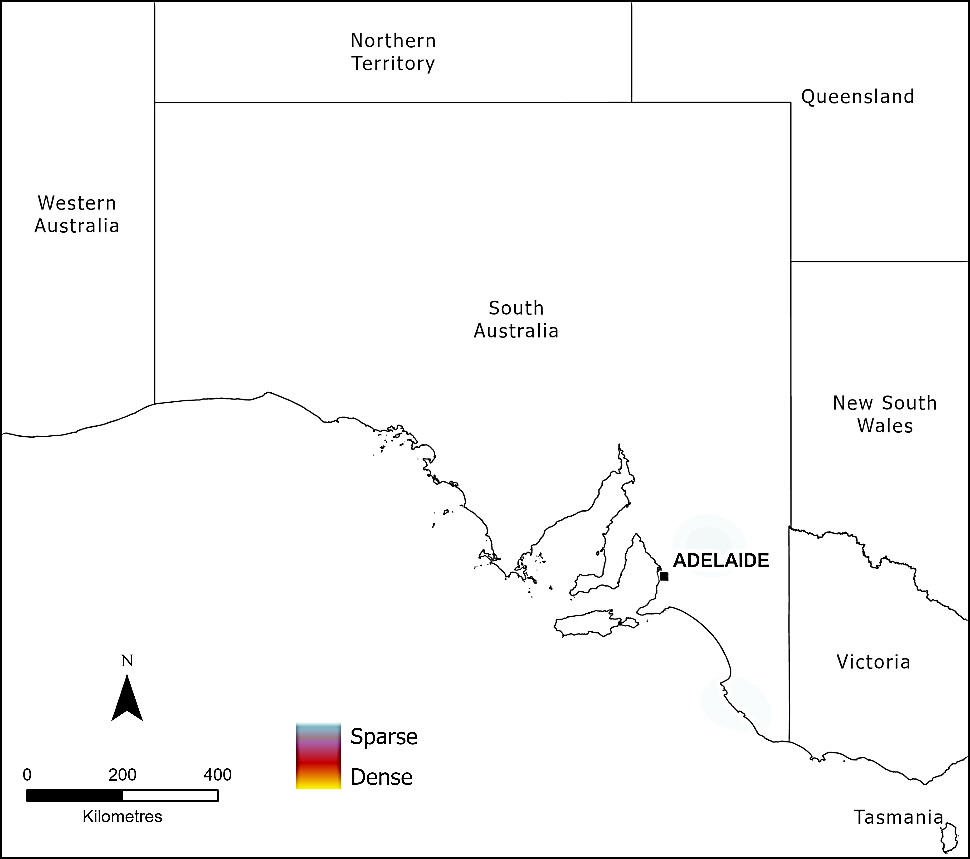


A

B


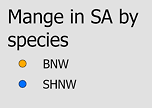


Supplementary Figure 7A. Map of bare-nosed (BNW) and southern hairy-nosed (SHNW) wombats in South Australia showing observable signs of sarcoptic mange. B. Heat map of bare-nosed wombats within SA showing observable signs of sarcoptic mange

Supplementary Figure 8. Seasonal distribution of sarcoptic mange in wombats reported to WomSAT between 2010 and 2024.


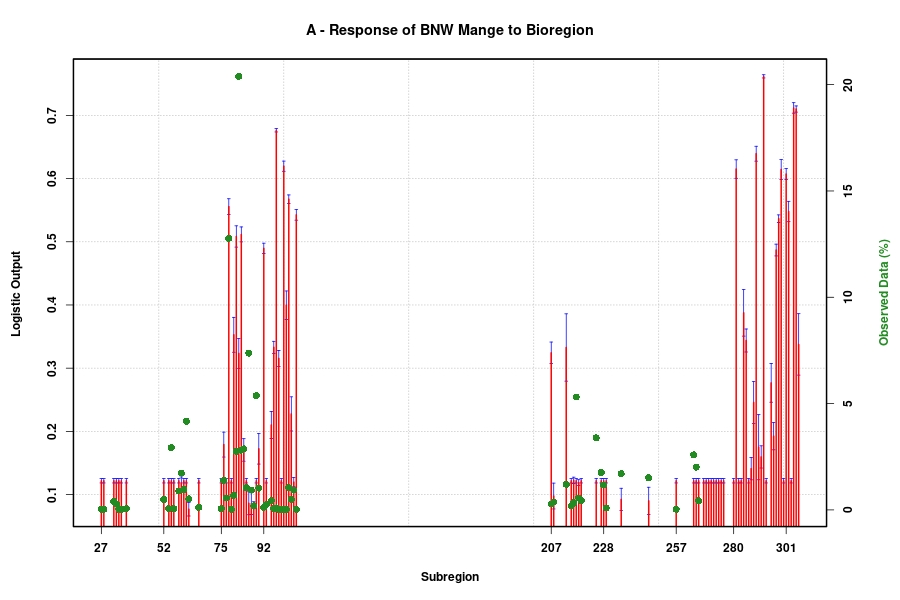


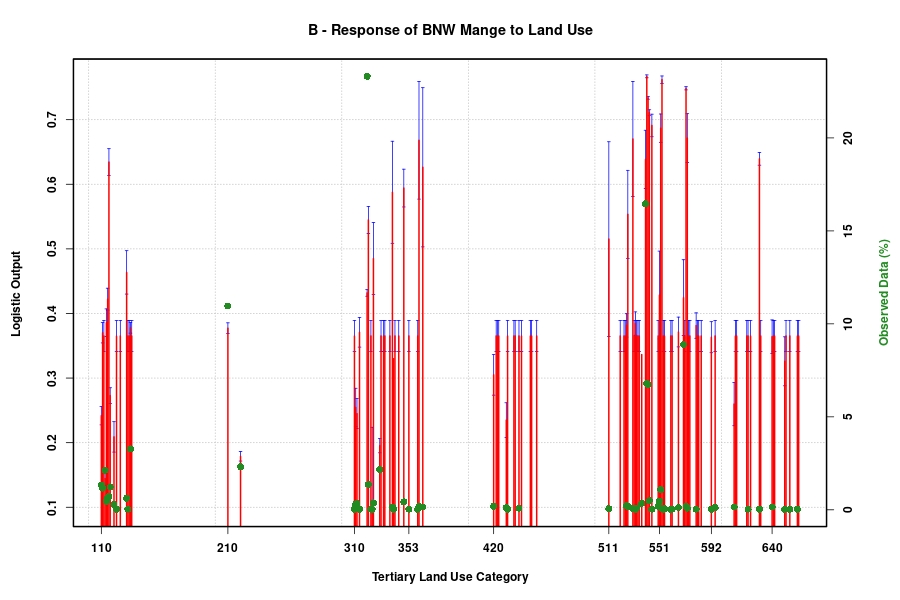


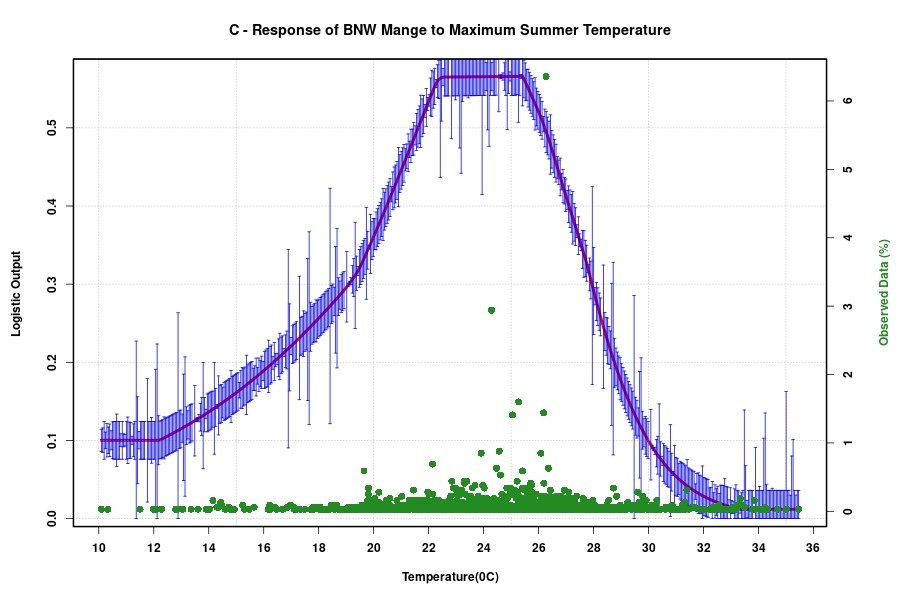

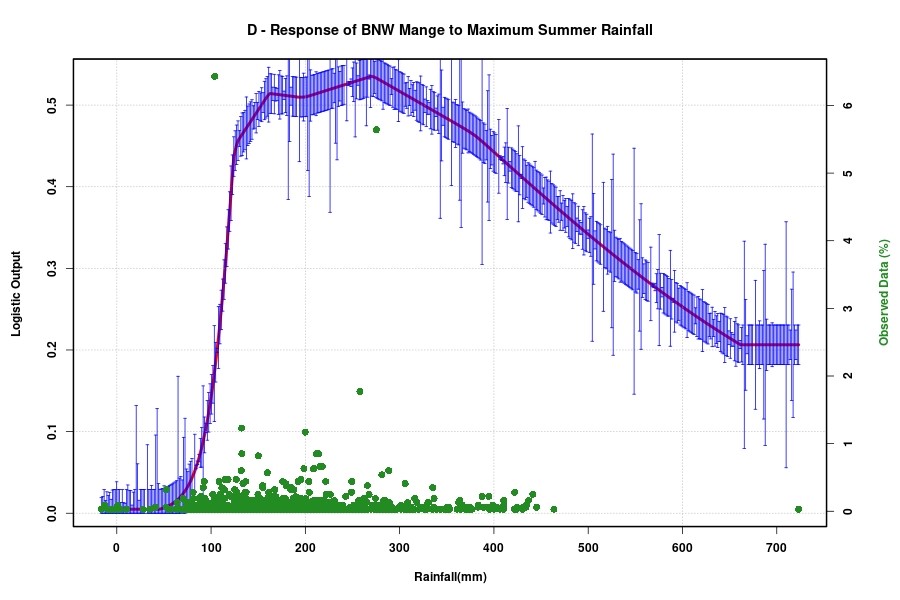

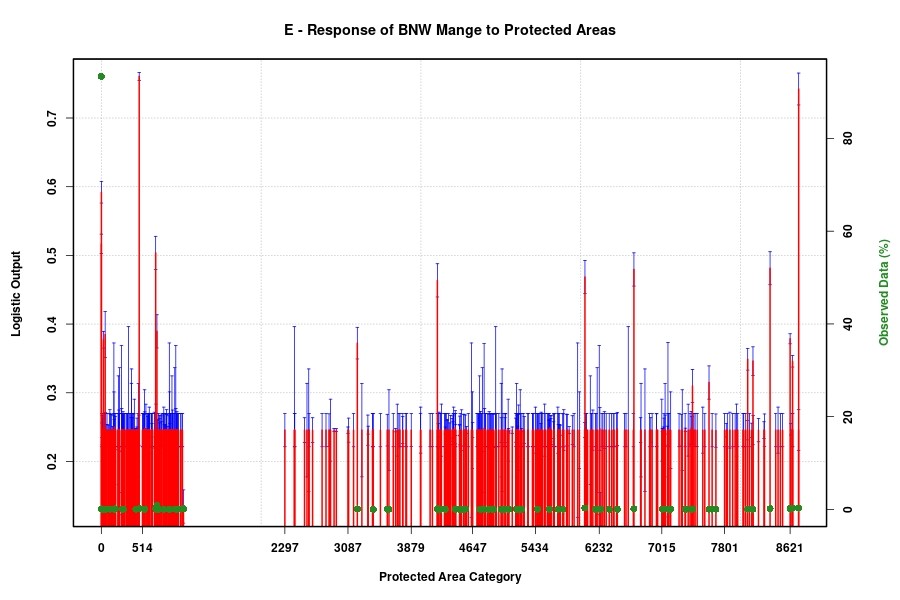

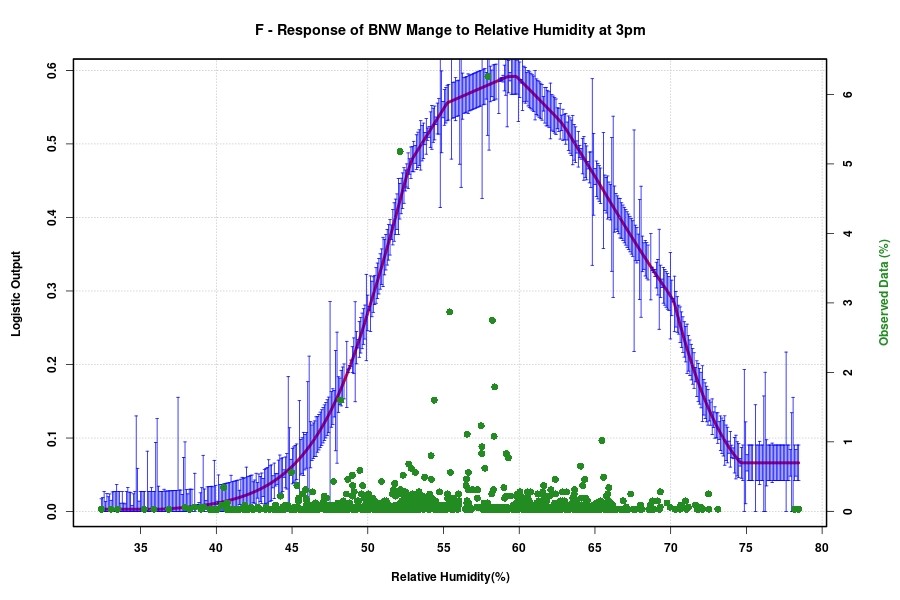


Supplementary Figure 9. Response of sarcoptic mange suitability infesting bare-nosed wombats for A, bioregion; B, land use; C, maximum summer temperature; D, rain in summer; E, protected areas and F, relative humidity at 3pm. Each graph shows the probability of mange presence across the range of each variable when reflecting the dependence of predicted suitability both on the selected variable and on dependencies induced by correlations between the selected variable and other variables. Red represents the mean of 15 model replicates and blue is the standard deviation. Predicted probabilities between 0.51 and 0.76 on the environmental factor graphs are suitable and probabilities above 0.77 are considered highly suitable for sarcoptic mange. Green dots represent observed data as a percentage.

Supplementary Table 1. Environmental layer description and classification of data type used to create the model of habitat suitability for sarcoptic mange occurrence in bare-nosed wombats.

| Name | Description | Type | Detailed description | Reference |
| --- | --- | --- | --- | --- |
| 90mready_clip_dem | Elevation (90m) | Continuous | Imported at a data resolution of 90m | (Gallant et al., 2009) |
| capad_nodata_to0_mask1 | Collaborative Australian Protected Areas Database | Categorical | Imported as a shape file and classified as conservation area, conservation covenant, conservation park, conservation reserve, game reserve, heritage agreement, heritage river, national park, nature conservation reserve, nature features reserve, nature recreation area, nature reserve, private sanctuary, regional reserve, remote and natural area, state conservation area and state park. | (Australian Government, 2022a) |
| clip_nvis06 | Vegetation type | Categorical | Collected as a shape file from L2 structural formation and classified as low woodland, low open woodland, mid open hammock grassland, mid open mallee woodland, mid open shrubland and tall sparse shrubland. | (Woollcombe, 2024) |
| clum_50m1220m | Catchment scale land use of Australia (tertiary land use) | Categorical | Downloaded as a shape file and classified as either grazing modified pastures, habitat/species management area, managed resource protection, national park, natural feature protection, nature conservation, other conserved area, other minimal use production native forests, protected landscape, strict nature reserves, recreation and culture, residential native cover, river, roads, urban residential, and utilities. | (ABARES, 2021) |
| distance_ahgf_minimum | Distance between water body and observation | Continuous | Utilised all water bodies in the layer which were classified as canal line, dam, hydro area, mapped stream and water body. | (Bureau of Meterology, 2024) |
| distance_aust_cities_osm_ pop200_mask | Distance between urban areas (population over 200) and observation | Continuous | Classified as cities with a population exceeding 200 people. The cutoff of 200 people was chosen as according to definition of urban localities according to the Australian Bureau of Statistics (ABS) (2021). | (OpenStreetMap, 2018) |
| distance_national_roads | Distance between roads and observation | Continuous | Utilised all road types in the layer including trails and unnamed roads | (DAA, 2024) |
| ibra | Interim Biogeographic Regionalisation of Australia | Categorical | Collected as a shape file and was overlayed on top of our presence only data. | (Australian Government, 2022b) |
| maxann | 19991-2020 Maximum annual temperature | Continuous |  | (Bureau of Meterology, 2022) |
| maxaut | 1991-2020 Maximum autumn temperature | Continuous |  | (Bureau of Meterology, 2022) |
| maxmaysep | 1991-2020 Maximum dry season temperature | Continuous |  | (Bureau of Meterology, 2022) |
| maxocapr | 1991-2020 Maximum wet season temperature | Continuous |  | (Bureau of Meterology, 2022) |
| maxspr | 1991-2020 Maximum spring temperature | Continuous |  | (Bureau of Meterology, 2022) |
| maxssum | 1991-2020 Maximum summer temperature | Continuous |  | (Bureau of Meterology, 2022) |
| maxwin | 1991-2020 Maximum winter temperature | Continuous |  | (Bureau of Meterology, 2022) |
| meanann | 1991-2020 Mean annual temperature | Continuous |  | (Bureau of Meterology, 2022) |
| meanaut | 1991-2020 Mean autumn temperature | Continuous |  | (Bureau of Meterology, 2022) |
| meanmaysep | 1991-2020 Mean dry season temperature | Continuous |  | (Bureau of Meterology, 2022) |
| meanoctapr | 1991-2020 Mean wet season temperature | Continuous |  | (Bureau of Meterology, 2022) |
| meanspr | 1991-2020 Mean spring temperature | Continuous |  | (Bureau of Meterology, 2022) |
| meansum | 1991-2020 Mean summer temperature | Continuous |  | (Bureau of Meterology, 2022) |
| meanwin | 1991-2020 Mean winter temperature | Continuous |  | (Bureau of Meterology, 2022) |
| minann | 1991-2020 Minimum annual temperature | Continuous |  | (Bureau of Meterology, 2022) |
| minaut | 1991-2020 Minimum autumn temperature | Continuous |  | (Bureau of Meterology, 2022) |
| minmaysep | 1991-2020 Minimum wet season temperature | Continuous |  | (Bureau of Meterology, 2022) |
| minocapr | 1991-2020 Minimum dry season temperature | Continuous |  | (Bureau of Meterology, 2022) |
| minspr | 1991-2020 Minimum spring temperature | Continuous |  | (Bureau of Meterology, 2022) |
| minsum | 1991-2020 Minimum summer temperature | Continuous |  | (Bureau of Meterology, 2022) |
| minwin | 1991-2020 Minimum winter temperature | Continuous |  | (Bureau of Meterology, 2022) |
| rainann | 1991-2020 Average annual rainfall | Continuous |  | (Bureau of Meterology, 2021) |
| rainaut | 1991-2020 Average autumn rainfall | Continuous |  | (Bureau of Meterology, 2021) |
| raindry | 1991-2020 Average dry season rainfall | Continuous |  | (Bureau of Meterology, 2021) |
| rainspr | 1991-2020 Average spring rainfall | Continuous |  | (Bureau of Meterology, 2021) |
| rainsum | 1991-2020 Average summer rainfall | Continuous |  | (Bureau of Meterology, 2021) |
| rainwet | 1991-2020 Average wet season rainfall | Continuous |  | (Bureau of Meterology, 2021) |
| rainwin | 1991-2020 Average winter rainfall | Continuous |  | (Bureau of Meterology, 2021) |
| rh9an1991_2020 | 1991-2020 Hourly relative humidity (9 am) | Continuous |  | (Bureau of Meterology, 2008) |
| rh15an1991_2020 | 1991-2020 Hourly relative humidity (3 pm) | Continuous |  | (Bureau of Meterology, 2008) |

Climatic factors were imported at a data resolution of 5km

Supplementary Table 2. Pearson coefficients for the environmental factors in the final bare-nosed wombat predictive presence model

|  | Elevation | Vegetation type | clum_50m1220m_resample | distance_ahgf_minimum | distance_national_roads | ibra | capad_nodata_to0 | climate_maxaut | climate_maxoctapr | climate_maxsum | climate_meanspr | climate_meanwin | climate_minmaysep | climate_minwin | climate_rainaut | climate_rainsum | climate_rainwet | climate_rainwin | rh15an1991_2020 | rh9an1991_2020 |
| --- | --- | --- | --- | --- | --- | --- | --- | --- | --- | --- | --- | --- | --- | --- | --- | --- | --- | --- | --- | --- |
| Elevation | 1 | 0 | 0 | 0 | 0 | 0 | 0 | 0 | 0 | 0 | 0 | 0 | 0 | 0 | 0 | 0 | 0 | 0 | 0 | 0 |
| Vegetation type | 0.003 | 1 | 0 | 0 | 0 | 0 | 0 | 0 | 0 | 0 | 0 | 0 | 0 | 0 | 0 | 0 | 0 | 0 | 0 | 0 |
| clum_50m1220m_resample | -0.135 | 0.307 | 1 | 0 | 0 | 0 | 0 | 0 | 0 | 0 | 0 | 0 | 0 | 0 | 0 | 0 | 0 | 0 | 0 | 0 |
| distance_ahgf_minimum | -0.113 | -0.062 | -0.175 | 1 | 0 | 0 | 0 | 0 | 0 | 0 | 0 | 0 | 0 | 0 | 0 | 0 | 0 | 0 | 0 | 0 |
| distance_national_roads | -0.124 | -0.214 | -0.093 | 0.315 | 1 | 0 | 0 | 0 | 0 | 0 | 0 | 0 | 0 | 0 | 0 | 0 | 0 | 0 | 0 | 0 |
| ibra | 0.166 | 0.063 | 0.034 | -0.306 | -0.206 | 1 | 0 | 0 | 0 | 0 | 0 | 0 | 0 | 0 | 0 | 0 | 0 | 0 | 0 | 0 |
| capad_nodata_to0 | 0.030 | -0.131 | -0.244 | 0.322 | 0.159 | -0.263 | 1 | 0 | 0 | 0 | 0 | 0 | 0 | 0 | 0 | 0 | 0 | 0 | 0 | 0 |
| climate_maxaut | -0.402 | -0.304 | -0.097 | 0.018 | 0.368 | 0.060 | -0.173 | 1 | 0 | 0 | 0 | 0 | 0 | 0 | 0 | 0 | 0 | 0 | 0 | 0 |
| climate_maxoctapr | -0.392 | -0.290 | -0.097 | 0.056 | 0.369 | -0.015 | -0.165 | 0.966 ** | 1 | 0 | 0 | 0 | 0 | 0 | 0 | 0 | 0 | 0 | 0 | 0 |
| climate_maxsum | -0.402 | -0.247 | -0.078 | 0.092 | 0.344 | -0.088 | -0.149 | 0.882 * | 0.973 ** | 1 | 0 | 0 | 0 | 0 | 0 | 0 | 0 | 0 | 0 | 0 |
| climate_meanspr | -0.358 | -0.347 | -0.111 | -0.033 | 0.361 | 0.098 | -0.166 | 0.982 ** | 0.916 ** | 0.803 * | 1 | 0 | 0 | 0 | 0 | 0 | 0 | 0 | 0 | 0 |
| climate_meanwin | -0.373 | -0.332 | -0.092 | -0.061 | 0.310 | 0.127 | -0.130 | 0.866 | 0.718 | 0.551 | 0.927 ** | 1 | 0 | 0 | 0 | 0 | 0 | 0 | 0 | 0 |
| climate_minmaysep | -0.39 | -0.326 | -0.068 | -0.095 | 0.269 | 0.117 | -0.106 | 0.766 | 0.600 | 0.428 | 0.853 * | 0.979 ** | 1 | 0 | 0 | 0 | 0 | 0 | 0 | 0 |
| climate_minwin | -0.393 | -0.315 | -0.061 | -0.092 | 0.250 | 0.105 | -0.09 | 0.713 | 0.537 | 0.361 | 0.806 * | 0.961 ** | 0.996 ** | 1 | 0 | 0 | 0 | 0 | 0 | 0 |
| climate_rainaut | 0.186 | -0.026 | -0.039 | -0.209 | -0.106 | 0.256 | 0.044 | -0.191 | -0.359 | -0.487 | -0.061 | 0.183 | 0.293 | 0.333 | 1 | 0 | 0 | 0 | 0 | 0 |
| climate_rainsum | 0.1 | -0.198 | -0.071 | -0.233 | 0.076 | 0.326 | -0.079 | 0.293 | 0.076 | -0.124 | 0.425 | 0.657 | 0.714 | 0.736 | 0.768 | 1 | 0 | 0 | 0 | 0 |
| climate_rainwet | 0.178 | -0.129 | -0.059 | -0.253 | -0.004 | 0.344 | -0.042 | 0.099 | -0.112 | -0.299 | 0.238 | 0.489 | 0.564 | 0.594 | 0.896 * | 0.968 ** | 1 | 0 | 0 | 0 |
| climate_rainwin | 0.340 | 0.248 | 0.090 | -0.122 | -0.299 | 0.059 | 0.19 | -0.808 * | -0.840 * | -0.823 * | -0.754 | -0.605 | -0.501 | -0.455 | 0.467 | -0.027 | 0.186 | 1 | 0 | 0 |
| rh15an1991_2020 | 0.169 | 0.182 | 0.079 | -0.117 | -0.266 | 0.186 | 0.094 | -0.611 | -0.784 | -0.88 * | -0.509 | -0.169 | -0.032 | 0.042 | 0.686 | 0.461 | 0.589 | 0.648 | 1 | 0 |
| rh9an1991_2020 | 0.274 | 0.244 | 0.113 | -0.163 | -0.357 | 0.252 | 0.045 | -0.672 | -0.814 | -0.883 * | -0.592 | -0.285 | -0.165 | -0.094 | 0.588 | 0.380 | 0.506 | 0.650 | 0.938 ** | 1 |

Note ** is a very strong correlation between variables, * is a strong correlation between variables. Detailed description of variables is provided in Supplementary Table 1

Supplementary Table 3. Predicted probability (% of total land area) for sarcoptic mange habitat suitability for local government areas (LGAs) within New South Wales, South Australia, Tasmania, Queensland and Victoria. LGAs that are “Moderately suitable” are highlighted in light pink, “suitable” in dark pink and “highly suitable” are highlighted purple. LGAs left unhighlighted are “not suitable” for sarcoptic mange.

| LGA name | LGA Code | State | Not suitable % | Moderately Suitable % | Suitable % | Highly Suitable % | Total area (square kilometres) |
| --- | --- | --- | --- | --- | --- | --- | --- |
| Albury | 10050 | NSW | 0.998489 | 0.001511 | 0 | 0 | 305.6386 |
| Armidale | 10180 | NSW | 0.909584 | 0.09041 | 6.22E-06 | 0 | 7809.441 |
| Ballina | 10250 | NSW | 1 | 0 | 0 | 0 | 484.9692 |
| Balranald | 10300 | NSW | 1 | 0 | 0 | 0 | 21690.75 |
| Bathurst | 10470 | NSW | 0.720326 | 0.273612 | 0.006062 | 0 | 3817.865 |
| Bayside (NSW) | 10500 | NSW | 0.250977 | 0.641554 | 0.107469 | 0 | 50.6204 |
| Bega Valley | 10550 | NSW | 0.617861 | 0.300901 | 0.081238 | 0 | 6278.501 |
| Bellingen | 10600 | NSW | 0.976172 | 0.023818 | 1.02E-05 | 0 | 1600.434 |
| Berrigan | 10650 | NSW | 1 | 0 | 0 | 0 | 2065.888 |
| Blacktown | 10750 | NSW | 0.044983 | 0.416039 | 0.538978 | 0 | 238.8471 |
| Bland | 10800 | NSW | 1 | 0 | 0 | 0 | 8557.654 |
| Blayney | 10850 | NSW | 0.907173 | 0.092567 | 0.00026 | 0 | 1524.647 |
| Blue Mountains | 10900 | NSW | 0.652341 | 0.181728 | 0.162671 | 0.00326 | 1431.144 |
| Bogan | 10950 | NSW | 1 | 0 | 0 | 0 | 14599.9 |
| Bourke | 11150 | NSW | 1 | 0 | 0 | 0 | 41598.37 |
| Brewarrina | 11200 | NSW | 1 | 0 | 0 | 0 | 19162.03 |
| Broken Hill | 11250 | NSW | 1 | 0 | 0 | 0 | 170.1153 |
| Burwood | 11300 | NSW | 0 | 0.143345 | 0.856655 | 0 | 7.1282 |
| Byron | 11350 | NSW | 0.976825 | 0.023175 | 0 | 0 | 565.8027 |
| Cabonne | 11400 | NSW | 0.994766 | 0.005234 | 0 | 0 | 6022.35 |
| Camden | 11450 | NSW | 0.004783 | 0.420803 | 0.574414 | 0 | 201.5278 |
| Campbelltown (NSW) | 11500 | NSW | 0.159152 | 0.520671 | 0.320177 | 0 | 311.4117 |
| Canada Bay | 11520 | NSW | 0.026675 | 0.797273 | 0.176052 | 0 | 19.9239 |
| Canterbury-Bankstown | 11570 | NSW | 0.031036 | 0.226079 | 0.742884 | 0 | 110.2374 |
| Carrathool | 11600 | NSW | 1 | 0 | 0 | 0 | 18934.49 |
| Central Coast (NSW) | 11650 | NSW | 0.544344 | 0.313348 | 0.142007 | 0.000301 | 1681.006 |
| Central Darling | 11700 | NSW | 1 | 0 | 0 | 0 | 53492.19 |
| Cessnock | 11720 | NSW | 0.434381 | 0.377946 | 0.186057 | 0.001616 | 1965.159 |
| Clarence Valley | 11730 | NSW | 0.986128 | 0.013849 | 2.27E-05 | 0 | 10428.68 |
| Cobar | 11750 | NSW | 1 | 0 | 0 | 0 | 45575.35 |
| Coffs Harbour | 11800 | NSW | 0.944853 | 0.055059 | 8.87E-05 | 0 | 1173.744 |
| Coolamon | 12000 | NSW | 1 | 0 | 0 | 0 | 2430.904 |
| Coonamble | 12150 | NSW | 1 | 0 | 0 | 0 | 9916.054 |
| Cootamundra-Gundagai | 12160 | NSW | 0.99999 | 1.02E-05 | 0 | 0 | 3981.417 |
| Cowra | 12350 | NSW | 0.999637 | 0.000363 | 0 | 0 | 2808.782 |
| Cumberland | 12380 | NSW | 0.001003 | 0.26738 | 0.731618 | 0 | 72.7496 |
| Dubbo | 12390 | NSW | 1 | 0 | 0 | 0 | 7534.525 |
| Dungog | 12700 | NSW | 0.713888 | 0.285864 | 0.000248 | 0 | 2249.998 |
| Edward River | 12730 | NSW | 1 | 0 | 0 | 0 | 8883.446 |
| Eurobodalla | 12750 | NSW | 0.744714 | 0.15811 | 0.097175 | 0 | 3428.17 |
| Fairfield | 12850 | NSW | 0.001834 | 0.291388 | 0.706778 | 0 | 101.5072 |
| Federation | 12870 | NSW | 1 | 0 | 0 | 0 | 5684.925 |
| Forbes | 12900 | NSW | 1 | 0 | 0 | 0 | 4710.128 |
| Georges River | 12930 | NSW | 0.011573 | 0.236191 | 0.752236 | 0 | 38.3375 |
| Gilgandra | 12950 | NSW | 1 | 0 | 0 | 0 | 4831.506 |
| Glen Innes Severn | 13010 | NSW | 0.983521 | 0.016479 | 0 | 0 | 5479.977 |
| Goulburn Mulwaree | 13310 | NSW | 0.247877 | 0.651696 | 0.100291 | 0.000136 | 3220.08 |
| Greater Hume | 13340 | NSW | 0.999414 | 0.000586 | 0 | 0 | 5749.388 |
| Griffith | 13450 | NSW | 1 | 0 | 0 | 0 | 1639.173 |
| Gunnedah | 13550 | NSW | 1 | 0 | 0 | 0 | 4987.03 |
| Gwydir | 13660 | NSW | 1 | 0 | 0 | 0 | 9259.654 |
| Hawkesbury | 13800 | NSW | 0.569467 | 0.319808 | 0.109085 | 0.00164 | 2775.138 |
| Hay | 13850 | NSW | 1 | 0 | 0 | 0 | 11325.93 |
| Hilltops | 13910 | NSW | 1 | 0 | 0 | 0 | 7140.887 |
| Hornsby | 14000 | NSW | 0.571388 | 0.259189 | 0.168853 | 0.00057 | 455.0374 |
| Hunters Hill | 14100 | NSW | 0.133858 | 0.57874 | 0.287402 | 0 | 5.7169 |
| Inner West | 14170 | NSW | 0.0412 | 0.600203 | 0.358596 | 0 | 35.2115 |
| Inverell | 14220 | NSW | 0.999965 | 3.53E-05 | 0 | 0 | 9404.756 |
| Junee | 14300 | NSW | 1 | 0 | 0 | 0 | 2030.022 |
| Kempsey | 14350 | NSW | 0.87742 | 0.122497 | 8.35E-05 | 0 | 3375.67 |
| Kiama | 14400 | NSW | 0.149258 | 0.248928 | 0.552788 | 0.049027 | 257.6638 |
| Ku-ring-gai | 14500 | NSW | 0.323766 | 0.341858 | 0.334375 | 0 | 85.4093 |
| Kyogle | 14550 | NSW | 0.995932 | 0.004068 | 0 | 0 | 3584.183 |
| Lachlan | 14600 | NSW | 1 | 0 | 0 | 0 | 14968.25 |
| Lake Macquarie | 14650 | NSW | 0.285349 | 0.440027 | 0.273975 | 0.00065 | 648.6393 |
| Lane Cove | 14700 | NSW | 0.158309 | 0.834808 | 0.006883 | 0 | 10.4789 |
| Leeton | 14750 | NSW | 1 | 0 | 0 | 0 | 1167.188 |
| Lismore | 14850 | NSW | 0.999092 | 0.000908 | 0 | 0 | 1287.704 |
| Lithgow | 14870 | NSW | 0.441158 | 0.325153 | 0.230861 | 0.002827 | 4512.256 |
| Liverpool | 14900 | NSW | 0.016399 | 0.584709 | 0.398893 | 0 | 305.7401 |
| Liverpool Plains | 14920 | NSW | 0.996943 | 0.003057 | 0 | 0 | 5082.24 |
| Lockhart | 14950 | NSW | 1 | 0 | 0 | 0 | 2895.805 |
| Maitland | 15050 | NSW | 0.362678 | 0.636473 | 0.000849 | 0 | 391.5089 |
| Mid-Coast | 15240 | NSW | 0.714089 | 0.283526 | 0.002384 | 0 | 10053.86 |
| Mid-Western | 15270 | NSW | 0.822714 | 0.156791 | 0.020484 | 1.11E-05 | 8752.259 |
| Moree Plains | 15300 | NSW | 1 | 0 | 0 | 0 | 17902.7 |
| Mosman | 15350 | NSW | 0.550376 | 0.449624 | 0 | 0 | 8.6503 |
| Murray River | 15520 | NSW | 1 | 0 | 0 | 0 | 11863.02 |
| Murrumbidgee | 15560 | NSW | 1 | 0 | 0 | 0 | 6880.79 |
| Muswellbrook | 15650 | NSW | 0.829251 | 0.16616 | 0.004589 | 0 | 3404.884 |
| Nambucca Valley | 15700 | NSW | 0.918301 | 0.0816 | 9.85E-05 | 0 | 1491.343 |
| Narrabri | 15750 | NSW | 1 | 0 | 0 | 0 | 13014.99 |
| Narrandera | 15800 | NSW | 1 | 0 | 0 | 0 | 4116.346 |
| Narromine | 15850 | NSW | 1 | 0 | 0 | 0 | 5261.517 |
| Newcastle | 15900 | NSW | 0.320682 | 0.561402 | 0.117188 | 0.000728 | 186.7255 |
| North Sydney | 15950 | NSW | 0.468566 | 0.531434 | 0 | 0 | 10.491 |
| Northern Beaches | 15990 | NSW | 0.616093 | 0.38146 | 0.002447 | 0 | 254.2072 |
| Oberon | 16100 | NSW | 0.650576 | 0.337776 | 0.011649 | 0 | 3625.047 |
| Orange | 16150 | NSW | 0.843153 | 0.156847 | 0 | 0 | 284.2159 |
| Parkes | 16200 | NSW | 1 | 0 | 0 | 0 | 5957.614 |
| Parramatta | 16260 | NSW | 0.055001 | 0.416868 | 0.52813 | 0 | 83.8303 |
| Penrith | 16350 | NSW | 0.096064 | 0.525442 | 0.378414 | 8E-05 | 404.7021 |
| Port Macquarie-Hastings | 16380 | NSW | 0.771592 | 0.226484 | 0.001924 | 0 | 3682.392 |
| Port Stephens | 16400 | NSW | 0.69512 | 0.304836 | 4.47E-05 | 0 | 858.4357 |
| Queanbeyan-Palerang | 16490 | NSW | 0.275637 | 0.657094 | 0.067268 | 0 | 5319.008 |
| Randwick | 16550 | NSW | 0.194665 | 0.805335 | 0 | 0 | 36.331 |
| Richmond Valley | 16610 | NSW | 0.999216 | 0.000784 | 0 | 0 | 3047.375 |
| Ryde | 16700 | NSW | 0.115573 | 0.563392 | 0.321035 | 0 | 40.4746 |
| Shellharbour | 16900 | NSW | 0.081572 | 0.112585 | 0.802044 | 0.003799 | 147.4208 |
| Shoalhaven | 16950 | NSW | 0.637749 | 0.230971 | 0.128222 | 0.003058 | 4567.201 |
| Singleton | 17000 | NSW | 0.642637 | 0.335929 | 0.021406 | 2.81E-05 | 4892.717 |
| Snowy Monaro | 17040 | NSW | 0.343811 | 0.52554 | 0.130596 | 5.29E-05 | 15164.79 |
| Snowy Valleys | 17080 | NSW | 0.993101 | 0.006891 | 8.14E-06 | 0 | 8958.922 |
| Strathfield | 17100 | NSW | 0 | 0.231391 | 0.768609 | 0 | 14.0008 |
| Sutherland | 17150 | NSW | 0.609982 | 0.375933 | 0.014085 | 0 | 333.5806 |
| Sydney | 17200 | NSW | 0.080026 | 0.912167 | 0.007807 | 0 | 26.6762 |
| Tamworth | 17310 | NSW | 0.995946 | 0.004054 | 0 | 0 | 9884.351 |
| Temora | 17350 | NSW | 1 | 0 | 0 | 0 | 2802.016 |
| Tenterfield | 17400 | NSW | 0.993452 | 0.006548 | 0 | 0 | 7324.307 |
| The Hills | 17420 | NSW | 0.021506 | 0.411427 | 0.564088 | 0.002979 | 386.1583 |
| Tweed | 17550 | NSW | 0.959852 | 0.039882 | 0.000267 | 0 | 1307.785 |
| Upper Hunter | 17620 | NSW | 0.943343 | 0.056622 | 3.5E-05 | 0 | 8096.089 |
| Upper Lachlan | 17640 | NSW | 0.853895 | 0.145175 | 0.00093 | 0 | 7127.396 |
| Uralla | 17650 | NSW | 0.995513 | 0.004487 | 0 | 0 | 3226.533 |
| Wagga Wagga | 17750 | NSW | 0.999955 | 4.53E-05 | 0 | 0 | 4824.489 |
| Walcha | 17850 | NSW | 0.988349 | 0.011651 | 0 | 0 | 6261.024 |
| Walgett | 17900 | NSW | 1 | 0 | 0 | 0 | 22308.25 |
| Warren | 17950 | NSW | 1 | 0 | 0 | 0 | 10753.77 |
| Warrumbungle | 18020 | NSW | 0.998165 | 0.001835 | 0 | 0 | 12372.12 |
| Waverley | 18050 | NSW | 0.083624 | 0.916376 | 0 | 0 | 9.3506 |
| Weddin | 18100 | NSW | 1 | 0 | 0 | 0 | 3414.857 |
| Wentworth | 18200 | NSW | 1 | 0 | 0 | 0 | 26255.93 |
| Willoughby | 18250 | NSW | 0.136799 | 0.605088 | 0.258112 | 0 | 22.4257 |
| Wingecarribee | 18350 | NSW | 0.303128 | 0.314856 | 0.377118 | 0.004897 | 2689.342 |
| Wollondilly | 18400 | NSW | 0.612106 | 0.23239 | 0.154712 | 0.000792 | 2555.434 |
| Wollongong | 18450 | NSW | 0.408548 | 0.388432 | 0.19164 | 0.011381 | 684.3076 |
| Yass Valley | 18710 | NSW | 0.905008 | 0.094867 | 0.000126 | 0 | 3995.017 |
| Unincorporated NSW | 19399 | NSW | 1 | 0 | 0 | 0 | 93208.92 |
| Alpine | 20110 | VIC | 0.891377 | 0.09429 | 0.014283 | 4.91E-05 | 4788.157 |
| Ararat | 20260 | VIC | 0.892097 | 0.106601 | 0.001302 | 0 | 4211.117 |
| Ballarat | 20570 | VIC | 0.72198 | 0.158401 | 0.11962 | 0 | 739.0321 |
| Banyule | 20660 | VIC | 0 | 0.473111 | 0.526889 | 0 | 62.5402 |
| Bass Coast | 20740 | VIC | 0.010228 | 0.66349 | 0.323491 | 0.002791 | 865.8094 |
| Baw Baw | 20830 | VIC | 0.475956 | 0.309975 | 0.210123 | 0.003946 | 4027.629 |
| Bayside (Vic.) | 20910 | VIC | 0 | 0.49221 | 0.50779 | 0 | 37.2111 |
| Benalla | 21010 | VIC | 0.770606 | 0.213084 | 0.01631 | 0 | 2352.64 |
| Boroondara | 21110 | VIC | 0.019259 | 0.383838 | 0.596902 | 0 | 60.1776 |
| Brimbank | 21180 | VIC | 0.452928 | 0.547072 | 0 | 0 | 123.3991 |
| Buloke | 21270 | VIC | 1 | 0 | 0 | 0 | 7998.247 |
| Campaspe | 21370 | VIC | 0.999996 | 3.59E-06 | 0 | 0 | 4519.073 |
| Cardinia | 21450 | VIC | 0.017676 | 0.489497 | 0.441529 | 0.051298 | 1282.567 |
| Casey | 21610 | VIC | 0.012637 | 0.374041 | 0.594641 | 0.018681 | 409.4287 |
| Central Goldfields | 21670 | VIC | 0.999789 | 0.000211 | 0 | 0 | 1532.784 |
| Colac Otway | 21750 | VIC | 0.619991 | 0.348366 | 0.03164 | 2.43E-06 | 3437.47 |
| Corangamite | 21830 | VIC | 0.710057 | 0.272727 | 0.017216 | 0 | 4407.492 |
| Darebin | 21890 | VIC | 0.003029 | 0.996062 | 0.000909 | 0 | 53.4712 |
| East Gippsland | 22110 | VIC | 0.802498 | 0.178538 | 0.018961 | 3.14E-06 | 20939.5 |
| Frankston | 22170 | VIC | 0.030076 | 0.284377 | 0.685547 | 0 | 129.5981 |
| Gannawarra | 22250 | VIC | 1 | 0 | 0 | 0 | 3737.598 |
| Glen Eira | 22310 | VIC | 0 | 0.746234 | 0.253766 | 0 | 38.6908 |
| Glenelg | 22410 | VIC | 0.938161 | 0.061535 | 0.000304 | 0 | 6218.788 |
| Golden Plains | 22490 | VIC | 0.675992 | 0.243327 | 0.080651 | 3E-05 | 2703.39 |
| Greater Bendigo | 22620 | VIC | 0.999897 | 0.000103 | 0 | 0 | 2999.972 |
| Greater Dandenong | 22670 | VIC | 0 | 0.279012 | 0.720988 | 0 | 129.5466 |
| Greater Geelong | 22750 | VIC | 0.59926 | 0.366944 | 0.033773 | 2.22E-05 | 1247.987 |
| Greater Shepparton | 22830 | VIC | 0.999997 | 3.34E-06 | 0 | 0 | 2421.909 |
| Hepburn | 22910 | VIC | 0.678764 | 0.237571 | 0.083665 | 0 | 1472.984 |
| Hindmarsh | 22980 | VIC | 1 | 0 | 0 | 0 | 7524.182 |
| Hobsons Bay | 23110 | VIC | 0.25661 | 0.74339 | 0 | 0 | 64.2405 |
| Horsham | 23190 | VIC | 0.999444 | 0.000556 | 0 | 0 | 4266.769 |
| Hume | 23270 | VIC | 0.589066 | 0.372118 | 0.038817 | 0 | 503.8488 |
| Indigo | 23350 | VIC | 0.945096 | 0.053604 | 0.0013 | 0 | 2040.471 |
| Kingston (Vic.) | 23430 | VIC | 0 | 0.119656 | 0.880344 | 0 | 91.3676 |
| Knox | 23670 | VIC | 0.000498 | 0.129 | 0.832243 | 0.038259 | 113.9114 |
| Latrobe (Vic.) | 23810 | VIC | 0.218621 | 0.551882 | 0.227104 | 0.002392 | 1425.613 |
| Loddon | 23940 | VIC | 1 | 0 | 0 | 0 | 6696.445 |
| Macedon Ranges | 24130 | VIC | 0.24284 | 0.539551 | 0.21721 | 0.000398 | 1748.284 |
| Manningham | 24210 | VIC | 0 | 0.210669 | 0.77982 | 0.009511 | 113.3479 |
| Mansfield | 24250 | VIC | 0.62629 | 0.274428 | 0.098711 | 0.000571 | 3843.854 |
| Maribyrnong | 24330 | VIC | 0.076383 | 0.923617 | 0 | 0 | 31.2258 |
| Maroondah | 24410 | VIC | 0 | 0.015441 | 0.974132 | 0.010426 | 61.4097 |
| Melbourne | 24600 | VIC | 0.000851 | 0.999149 | 0 | 0 | 37.5452 |
| Melton | 24650 | VIC | 0.772946 | 0.211918 | 0.015121 | 1.54E-05 | 527.5374 |
| Merri-bek | 24700 | VIC | 0.002062 | 0.997938 | 0 | 0 | 50.9522 |
| Mildura | 24780 | VIC | 1 | 0 | 0 | 0 | 22081.92 |
| Mitchell | 24850 | VIC | 0.524137 | 0.39973 | 0.075368 | 0.000764 | 2862.084 |
| Moira | 24900 | VIC | 1 | 0 | 0 | 0 | 4045.92 |
| Monash | 24970 | VIC | 0 | 0.009845 | 0.990155 | 0 | 81.4829 |
| Moonee Valley | 25060 | VIC | 0.003016 | 0.996984 | 0 | 0 | 43.1384 |
| Moorabool | 25150 | VIC | 0.535481 | 0.32748 | 0.136682 | 0.000357 | 2110.55 |
| Mornington Peninsula | 25340 | VIC | 0.051858 | 0.598657 | 0.349485 | 0 | 723.992 |
| Mount Alexander | 25430 | VIC | 0.9466 | 0.052484 | 0.000916 | 0 | 1529.618 |
| Moyne | 25490 | VIC | 0.839047 | 0.155665 | 0.005286 | 1.51E-06 | 5481.686 |
| Murrindindi | 25620 | VIC | 0.426659 | 0.394495 | 0.176251 | 0.002595 | 3879.751 |
| Nillumbik | 25710 | VIC | 0.002117 | 0.208265 | 0.775832 | 0.013787 | 432.3373 |
| Northern Grampians | 25810 | VIC | 0.994973 | 0.004946 | 8.06E-05 | 0 | 5729.877 |
| Port Phillip | 25900 | VIC | 0 | 1 | 0 | 0 | 20.6141 |
| Pyrenees | 25990 | VIC | 0.867793 | 0.120767 | 0.011438 | 2.36E-06 | 3434.569 |
| South Gippsland | 26170 | VIC | 0.126831 | 0.449045 | 0.420312 | 0.003812 | 3296.331 |
| Southern Grampians | 26260 | VIC | 0.981521 | 0.018431 | 4.75E-05 | 0 | 6654.019 |
| Stonnington | 26350 | VIC | 0 | 0.787312 | 0.212688 | 0 | 25.651 |
| Strathbogie | 26430 | VIC | 0.689061 | 0.296738 | 0.014201 | 0 | 3303.257 |
| Surf Coast | 26490 | VIC | 0.62336 | 0.362582 | 0.014058 | 0 | 1552.944 |
| Swan Hill | 26610 | VIC | 1 | 0 | 0 | 0 | 6115.537 |
| Towong | 26670 | VIC | 0.928236 | 0.068025 | 0.003739 | 0 | 6675.173 |
| Wangaratta | 26700 | VIC | 0.790044 | 0.183291 | 0.026554 | 0.000111 | 3645.11 |
| Warrnambool | 26730 | VIC | 0.504359 | 0.407381 | 0.08826 | 0 | 120.9487 |
| Wellington | 26810 | VIC | 0.550062 | 0.399916 | 0.049756 | 0.000266 | 10817.42 |
| West Wimmera | 26890 | VIC | 0.999986 | 1.42E-05 | 0 | 0 | 9108.679 |
| Whitehorse | 26980 | VIC | 0 | 0.013468 | 0.986532 | 0 | 64.2801 |
| Whittlesea | 27070 | VIC | 0.315946 | 0.454779 | 0.227257 | 0.002018 | 489.6938 |
| Wodonga | 27170 | VIC | 0.969105 | 0.030895 | 0 | 0 | 433.0021 |
| Wyndham | 27260 | VIC | 0.690584 | 0.309416 | 0 | 0 | 542.0943 |
| Yarra | 27350 | VIC | 0.087967 | 0.894191 | 0.017842 | 0 | 19.5421 |
| Yarra Ranges | 27450 | VIC | 0.556586 | 0.074457 | 0.318277 | 0.05068 | 2468.205 |
| Yarriambiack | 27630 | VIC | 1 | 0 | 0 | 0 | 7325.797 |
| Unincorporated Vic | 29399 | VIC | 0.317265 | 0.627954 | 0.054782 | 0 | 283.1903 |
| Adelaide | 40070 | SA | 1 | 0 | 0 | 0 | 15.5733 |
| Adelaide Hills | 40120 | SA | 1 | 0 | 0 | 0 | 794.4957 |
| Adelaide Plains | 40150 | SA | 1 | 0 | 0 | 0 | 932.4913 |
| Alexandrina | 40220 | SA | 1 | 0 | 0 | 0 | 1826.807 |
| Anangu Pitjantjatjara Yankunytjatjara | 40250 | SA | 1 | 0 | 0 | 0 | 102348.1 |
| Barossa | 40310 | SA | 1 | 0 | 0 | 0 | 893.5424 |
| Barunga West | 40430 | SA | 1 | 0 | 0 | 0 | 1590.389 |
| Berri Barmera | 40520 | SA | 1 | 0 | 0 | 0 | 476.1962 |
| Burnside | 40700 | SA | 1 | 0 | 0 | 0 | 27.5183 |
| Campbelltown (SA) | 40910 | SA | 1 | 0 | 0 | 0 | 24.3485 |
| Ceduna | 41010 | SA | 1 | 0 | 0 | 0 | 5420.344 |
| Charles Sturt | 41060 | SA | 1 | 0 | 0 | 0 | 54.7876 |
| Clare and Gilbert Valleys | 41140 | SA | 1 | 0 | 0 | 0 | 1892.447 |
| Cleve | 41190 | SA | 1 | 0 | 0 | 0 | 5018.848 |
| Coober Pedy | 41330 | SA | 1 | 0 | 0 | 0 | 77.6795 |
| Copper Coast | 41560 | SA | 1 | 0 | 0 | 0 | 772.9149 |
| Elliston | 41750 | SA | 1 | 0 | 0 | 0 | 6741.932 |
| Flinders Ranges | 41830 | SA | 1 | 0 | 0 | 0 | 4070.483 |
| Franklin Harbour | 41960 | SA | 1 | 0 | 0 | 0 | 2755.564 |
| Gawler | 42030 | SA | 1 | 0 | 0 | 0 | 41.1379 |
| Goyder | 42110 | SA | 1 | 0 | 0 | 0 | 6715.142 |
| Grant | 42250 | SA | 0.97794 | 0.02206 | 0 | 0 | 1897.824 |
| Holdfast Bay | 42600 | SA | 1 | 0 | 0 | 0 | 13.752 |
| Kangaroo Island | 42750 | SA | 1 | 0 | 0 | 0 | 4400.863 |
| Karoonda East Murray | 43080 | SA | 1 | 0 | 0 | 0 | 4415.796 |
| Kimba | 43220 | SA | 1 | 0 | 0 | 0 | 5697.107 |
| Kingston (SA) | 43360 | SA | 1 | 0 | 0 | 0 | 3339.795 |
| Light | 43650 | SA | 1 | 0 | 0 | 0 | 1276.845 |
| Lower Eyre Peninsula | 43710 | SA | 1 | 0 | 0 | 0 | 4721.392 |
| Loxton Waikerie | 43790 | SA | 1 | 0 | 0 | 0 | 7763.871 |
| Maralinga Tjarutja | 44000 | SA | 1 | 0 | 0 | 0 | 105766.3 |
| Marion | 44060 | SA | 1 | 0 | 0 | 0 | 55.6406 |
| Mid Murray | 44210 | SA | 1 | 0 | 0 | 0 | 6271.626 |
| Mitcham | 44340 | SA | 1 | 0 | 0 | 0 | 75.5468 |
| Mount Barker | 44550 | SA | 1 | 0 | 0 | 0 | 594.6406 |
| Mount Gambier | 44620 | SA | 0.437634 | 0.562366 | 0 | 0 | 33.8938 |
| Mount Remarkable | 44830 | SA | 1 | 0 | 0 | 0 | 3422.773 |
| Murray Bridge | 45040 | SA | 1 | 0 | 0 | 0 | 1831.76 |
| Naracoorte Lucindale | 45090 | SA | 0.999996 | 3.58E-06 | 0 | 0 | 4519.715 |
| Northern Areas | 45120 | SA | 1 | 0 | 0 | 0 | 2986.182 |
| Norwood Payneham and St Peters | 45290 | SA | 1 | 0 | 0 | 0 | 15.1036 |
| Onkaparinga | 45340 | SA | 1 | 0 | 0 | 0 | 518.1255 |
| Orroroo Carrieton | 45400 | SA | 1 | 0 | 0 | 0 | 3321.824 |
| Peterborough | 45540 | SA | 1 | 0 | 0 | 0 | 3020.142 |
| Playford | 45680 | SA | 1 | 0 | 0 | 0 | 345.2038 |
| Port Adelaide Enfield | 45890 | SA | 1 | 0 | 0 | 0 | 91.7588 |
| Port Augusta | 46090 | SA | 1 | 0 | 0 | 0 | 1157.991 |
| Port Lincoln | 46300 | SA | 1 | 0 | 0 | 0 | 30.3681 |
| Port Pirie | 46450 | SA | 1 | 0 | 0 | 0 | 1760.71 |
| Prospect | 46510 | SA | 1 | 0 | 0 | 0 | 7.7915 |
| Renmark Paringa | 46670 | SA | 1 | 0 | 0 | 0 | 916.442 |
| Robe | 46860 | SA | 1 | 0 | 0 | 0 | 1091.716 |
| Roxby Downs | 46970 | SA | 1 | 0 | 0 | 0 | 110.4778 |
| Salisbury | 47140 | SA | 1 | 0 | 0 | 0 | 159.8432 |
| Southern Mallee | 47290 | SA | 1 | 0 | 0 | 0 | 5702.202 |
| Streaky Bay | 47490 | SA | 1 | 0 | 0 | 0 | 6222.859 |
| Tatiara | 47630 | SA | 1 | 0 | 0 | 0 | 6527.234 |
| Tea Tree Gully | 47700 | SA | 1 | 0 | 0 | 0 | 95.2109 |
| Coorong | 47800 | SA | 1 | 0 | 0 | 0 | 8833.434 |
| Tumby Bay | 47910 | SA | 1 | 0 | 0 | 0 | 2669.079 |
| Unley | 47980 | SA | 1 | 0 | 0 | 0 | 14.2703 |
| Victor Harbor | 48050 | SA | 1 | 0 | 0 | 0 | 384.6255 |
| Wakefield | 48130 | SA | 1 | 0 | 0 | 0 | 3468.488 |
| Walkerville | 48260 | SA | 1 | 0 | 0 | 0 | 3.53 |
| Wattle Range | 48340 | SA | 0.996674 | 0.003326 | 0 | 0 | 3926.21 |
| West Torrens | 48410 | SA | 1 | 0 | 0 | 0 | 37.0886 |
| Whyalla | 48540 | SA | 1 | 0 | 0 | 0 | 1071.34 |
| Wudinna | 48640 | SA | 1 | 0 | 0 | 0 | 5075.278 |
| Yankalilla | 48750 | SA | 1 | 0 | 0 | 0 | 751.2577 |
| Yorke Peninsula | 48830 | SA | 1 | 0 | 0 | 0 | 5899.64 |
| Unincorporated SA | 49399 | SA | 1 | 0 | 0 | 0 | 619426.1 |
| Break O'Day | 60210 | TAS | 0.677745 | 0.279664 | 0.041926 | 0.000664 | 3523.886 |
| Brighton | 60410 | TAS | 0.019972 | 0.597564 | 0.380503 | 0.001961 | 171.1508 |
| Burnie | 60610 | TAS | 0.729475 | 0.234789 | 0.035695 | 4.07E-05 | 610.9528 |
| Central Coast (Tas.) | 60810 | TAS | 0.513812 | 0.43281 | 0.053252 | 0.000126 | 933.0891 |
| Central Highlands (Tas.) | 61010 | TAS | 0.671222 | 0.303242 | 0.025395 | 0.000141 | 7982.416 |
| Circular Head | 61210 | TAS | 0.973906 | 0.02588 | 0.000214 | 0 | 4897.651 |
| Clarence | 61410 | TAS | 0.042462 | 0.644229 | 0.309974 | 0.003334 | 377.9614 |
| Derwent Valley | 61510 | TAS | 0.893447 | 0.084048 | 0.022345 | 0.00016 | 4108.111 |
| Devonport | 61610 | TAS | 0.039042 | 0.609986 | 0.347907 | 0.003065 | 111.259 |
| Dorset | 61810 | TAS | 0.535711 | 0.44811 | 0.016106 | 7.31E-05 | 3230.546 |
| Flinders (Tas.) | 62010 | TAS | 0.210587 | 0.760037 | 0.029377 | 0 | 1996.624 |
| George Town | 62210 | TAS | 0.345053 | 0.613157 | 0.041607 | 0.000183 | 653.4308 |
| Glamorgan-Spring Bay | 62410 | TAS | 0.254837 | 0.574898 | 0.168141 | 0.002124 | 2591.591 |
| Glenorchy | 62610 | TAS | 0.386375 | 0.240102 | 0.362883 | 0.010641 | 121.1632 |
| Hobart | 62810 | TAS | 0.35323 | 0.274666 | 0.371797 | 0.000307 | 77.8965 |
| Huon Valley | 63010 | TAS | 0.857537 | 0.116112 | 0.026175 | 0.000176 | 5507.354 |
| Kentish | 63210 | TAS | 0.561949 | 0.390886 | 0.046548 | 0.000616 | 1156.189 |
| King Island | 63410 | TAS | 0.844948 | 0.155035 | 1.65E-05 | 0 | 1095.741 |
| Kingborough | 63610 | TAS | 0.28137 | 0.452342 | 0.263347 | 0.00294 | 720.1093 |
| Latrobe (Tas.) | 63810 | TAS | 0.430465 | 0.504976 | 0.064527 | 3.19E-05 | 600.4619 |
| Launceston | 64010 | TAS | 0.632237 | 0.266227 | 0.100759 | 0.000776 | 1413.631 |
| Meander Valley | 64210 | TAS | 0.59453 | 0.342393 | 0.062858 | 0.000219 | 3330.815 |
| Northern Midlands | 64610 | TAS | 0.343502 | 0.62106 | 0.0354 | 3.79E-05 | 5135.251 |
| Sorell | 64810 | TAS | 0.184416 | 0.62377 | 0.191687 | 0.000126 | 583.7723 |
| Southern Midlands | 65010 | TAS | 0.231631 | 0.720349 | 0.048011 | 9.29E-06 | 2615.494 |
| Tasman | 65210 | TAS | 0.471429 | 0.413422 | 0.113183 | 0.001967 | 660.3947 |
| Waratah-Wynyard | 65410 | TAS | 0.859925 | 0.125273 | 0.014756 | 4.61E-05 | 3535.908 |
| West Coast | 65610 | TAS | 0.985295 | 0.014471 | 0.00023 | 3.53E-06 | 9583.539 |
| West Tamar | 65810 | TAS | 0.255609 | 0.557589 | 0.185775 | 0.001027 | 691.1537 |
| Unincorporated ACT | 89399 | ACT | 0.688555 | 0.309827 | 0.001618 | 0 | 2358.133 |
| Aurukun | 30250 | QLD | 1 | 0 | 0 | 0 | 7423.989 |
| Balonne | 30300 | QLD | 1 | 0 | 0 | 0 | 31104.09 |
| Banana | 30370 | QLD | 1 | 0 | 0 | 0 | 28550.16 |
| Barcaldine | 30410 | QLD | 1 | 0 | 0 | 0 | 53382.65 |
| Barcoo | 30450 | QLD | 1 | 0 | 0 | 0 | 61830.17 |
| Blackall Tambo | 30760 | QLD | 1 | 0 | 0 | 0 | 30537.25 |
| Boulia | 30900 | QLD | 1 | 0 | 0 | 0 | 60906.47 |
| Brisbane | 31000 | QLD | 1 | 0 | 0 | 0 | 1342.717 |
| Bulloo | 31750 | QLD | 1 | 0 | 0 | 0 | 73722.01 |
| Bundaberg | 31820 | QLD | 1 | 0 | 0 | 0 | 6431.089 |
| Burdekin | 31900 | QLD | 0.999886 | 0.000114 | 0 | 0 | 5043.932 |
| Burke | 31950 | QLD | 1 | 0 | 0 | 0 | 39681.22 |
| Cairns | 32080 | QLD | 1 | 0 | 0 | 0 | 1689.323 |
| Carpentaria | 32250 | QLD | 1 | 0 | 0 | 0 | 64121.01 |
| Cassowary Coast | 32260 | QLD | 1 | 0 | 0 | 0 | 4687.824 |
| Central Highlands (Qld) | 32270 | QLD | 1 | 0 | 0 | 0 | 59834.84 |
| Charters Towers | 32310 | QLD | 1 | 0 | 0 | 0 | 68382.16 |
| Cherbourg | 32330 | QLD | 1 | 0 | 0 | 0 | 31.6002 |
| Cloncurry | 32450 | QLD | 1 | 0 | 0 | 0 | 47971.26 |
| Cook | 32500 | QLD | 0.997397 | 0.002603 | 0 | 0 | 105717.8 |
| Croydon | 32600 | QLD | 1 | 0 | 0 | 0 | 29497.9 |
| Diamantina | 32750 | QLD | 1 | 0 | 0 | 0 | 94722.43 |
| Doomadgee | 32770 | QLD | 1 | 0 | 0 | 0 | 1827.652 |
| Douglas | 32810 | QLD | 1 | 0 | 0 | 0 | 2427.863 |
| Etheridge | 33100 | QLD | 1 | 0 | 0 | 0 | 39199.3 |
| Flinders (Qld) | 33200 | QLD | 1 | 0 | 0 | 0 | 41199.94 |
| Fraser Coast | 33220 | QLD | 1 | 0 | 0 | 0 | 7104.598 |
| Gladstone | 33360 | QLD | 0.999995 | 4.83E-06 | 0 | 0 | 10484.29 |
| Gold Coast | 33430 | QLD | 0.991721 | 0.008279 | 0 | 0 | 1333.386 |
| Goondiwindi | 33610 | QLD | 1 | 0 | 0 | 0 | 19265.71 |
| Gympie | 33620 | QLD | 0.99974 | 0.00026 | 0 | 0 | 6883.978 |
| Hinchinbrook | 33800 | QLD | 1 | 0 | 0 | 0 | 2807.189 |
| Hope Vale | 33830 | QLD | 1 | 0 | 0 | 0 | 1111.71 |
| Ipswich | 33960 | QLD | 0.986669 | 0.013331 | 0 | 0 | 1093.856 |
| Isaac | 33980 | QLD | 1 | 0 | 0 | 0 | 58707.92 |
| Kowanyama | 34420 | QLD | 1 | 0 | 0 | 0 | 2555.333 |
| Livingstone | 34530 | QLD | 1 | 0 | 0 | 0 | 11757.87 |
| Lockhart River | 34570 | QLD | 0.997706 | 0.002294 | 0 | 0 | 3576.684 |
| Lockyer Valley | 34580 | QLD | 0.97587 | 0.02413 | 0 | 0 | 2269.005 |
| Logan | 34590 | QLD | 0.999121 | 0.000879 | 0 | 0 | 958.1333 |
| Longreach | 34710 | QLD | 1 | 0 | 0 | 0 | 40572.24 |
| Mackay | 34770 | QLD | 1 | 0 | 0 | 0 | 7613.296 |
| McKinlay | 34800 | QLD | 1 | 0 | 0 | 0 | 40736.8 |
| Mapoon | 34830 | QLD | 0.99817 | 0.00183 | 0 | 0 | 537.2366 |
| Maranoa | 34860 | QLD | 1 | 0 | 0 | 0 | 58719.34 |
| Mareeba | 34880 | QLD | 0.999853 | 0.000147 | 0 | 0 | 53491.05 |
| Moreton Bay | 35010 | QLD | 0.994417 | 0.005583 | 0 | 0 | 2041.474 |
| Mornington | 35250 | QLD | 1 | 0 | 0 | 0 | 1247.693 |
| Mount Isa | 35300 | QLD | 1 | 0 | 0 | 0 | 43715.2 |
| Murweh | 35600 | QLD | 1 | 0 | 0 | 0 | 40699.76 |
| Napranum | 35670 | QLD | 0.99798 | 0.00202 | 0 | 0 | 2004.347 |
| Noosa | 35740 | QLD | 1 | 0 | 0 | 0 | 869.8657 |
| North Burnett | 35760 | QLD | 0.999954 | 4.61E-05 | 0 | 0 | 19669.85 |
| Northern Peninsula Area | 35780 | QLD | 0.740251 | 0.259749 | 0 | 0 | 1052.068 |
| Palm Island | 35790 | QLD | 1 | 0 | 0 | 0 | 71.9928 |
| Paroo | 35800 | QLD | 1 | 0 | 0 | 0 | 47614.92 |
| Pormpuraaw | 36070 | QLD | 1 | 0 | 0 | 0 | 4395.365 |
| Quilpie | 36150 | QLD | 1 | 0 | 0 | 0 | 67414.66 |
| Redland | 36250 | QLD | 1 | 0 | 0 | 0 | 537.1672 |
| Richmond | 36300 | QLD | 1 | 0 | 0 | 0 | 26580.75 |
| Rockhampton | 36370 | QLD | 1 | 0 | 0 | 0 | 6570.267 |
| Scenic Rim | 36510 | QLD | 0.953154 | 0.046702 | 0.000143 | 0 | 4243.001 |
| Somerset | 36580 | QLD | 0.999974 | 2.56E-05 | 0 | 0 | 5373.434 |
| South Burnett | 36630 | QLD | 1 | 0 | 0 | 0 | 8381.558 |
| Southern Downs | 36660 | QLD | 0.998795 | 0.001205 | 0 | 0 | 7106.436 |
| Sunshine Coast | 36720 | QLD | 1 | 0 | 0 | 0 | 2253.852 |
| Tablelands | 36820 | QLD | 0.999999 | 7.17E-07 | 0 | 0 | 11292.87 |
| Toowoomba | 36910 | QLD | 0.999815 | 0.000185 | 0 | 0 | 12957.21 |
| Torres | 36950 | QLD | 0.752748 | 0.247252 | 0 | 0 | 883.7402 |
| Torres Strait Island | 36960 | QLD | 0.688913 | 0.311087 | 0 | 0 | 490.0453 |
| Townsville | 37010 | QLD | 0.999744 | 0.000256 | 0 | 0 | 3730.82 |
| Weipa | 37300 | QLD | 0.884956 | 0.115044 | 0 | 0 | 10.4643 |
| Western Downs | 37310 | QLD | 1 | 0 | 0 | 0 | 37922.5 |
| Whitsunday | 37340 | QLD | 0.99974 | 0.00026 | 0 | 0 | 23818.97 |
| Winton | 37400 | QLD | 1 | 0 | 0 | 0 | 53813.48 |
| Woorabinda | 37550 | QLD | 1 | 0 | 0 | 0 | 390.5578 |
| Wujal Wujal | 37570 | QLD | 1 | 0 | 0 | 0 | 11.842 |
| Yarrabah | 37600 | QLD | 1 | 0 | 0 | 0 | 158.8381 |

Note: The suitability levels recorded are the highest level recorded in a cell in a given LGA

**References**

ABARES. (2021). *Catchment Scale Land Use of Australia – Update December 2020*. Retrieved from: <https://www.agriculture.gov.au/abares/aclump/catchment-scale-land-use-of-australia-update-december-2020>

Australian Government. (2022a). *CAPAD 2022*. Retrieved from: <https://www.dcceew.gov.au/environment/land/nrs/science/ibra#daff-page-main>

Australian Government. (2022b). *IBRA7*. Retrieved from: <https://www.dcceew.gov.au/environment/land/nrs/science/ibra#daff-page-main>

Bureau of Meterology. (2008). *Relative humidity: Average 9 am and 3 pm relative humidity*. Retrieved from: <http://www.bom.gov.au/climate/maps/averages/relative-humidity/>

Bureau of Meterology. (2021). *Rainfall: Average annual, seasonal and monthly rainfall maps*. Retrieved from: <http://www.bom.gov.au/climate/maps/averages/rainfall/>

Bureau of Meterology. (2022). *Temperature: Average monthly and annual temperature maps*. Retrieved from: <http://www.bom.gov.au/climate/maps/averages/temperature/>

Bureau of Meterology. (2024). *Australian hydrological geospatial fabric (Geofabric)*. Retrieved from: <http://www.bom.gov.au/water/geofabric/>

DAA. (2024). *National roads*. Retrieved from: <https://digital.atlas.gov.au/datasets/3128b5993f4544b287779afbe76f3d72/explore?location=-27.509507%2C135.578063%2C5.74>

Gallant, J., Wilson, N., Tickle, P. K., Dowling, T., & Read, A. (2009). *3 second SRTM Derived Digital Elevation Model (DEM)*. Retrieved from: <https://pid.geoscience.gov.au/dataset/ga/69888>

OpenStreetMap. (2018). *Australia and Oceania [Australia]*. Retrieved from: <https://download.geofabrik.de/australia-oceania.html>

Woollcombe, D. (2024). *National vegetation information system (nvis) version 6.0 - australia vectors*. Retrieved from: <https://fed.dcceew.gov.au/datasets/national-vegetation-information-system-nvis-version-6-0-australia-vectors/about>
